# Atomistic Insights into Oxidation Mechanisms in Chemical–Mechanical Polishing of Diamond

**DOI:** 10.3390/mi17101107

**Published:** 2026-09-22

**Authors:** Kechong Wang, Yunkai Wang, Meng Li, Xiang Li, Chongjun Wu, Suye Hao, Ningchang Wang, Chen Li

**Affiliations:** 1 School of Mechatronics Engineering, Harbin Institute of Technology, Harbin 150001, China; charles_wkc@163.com (K.W.); 2024110129@stu.hit.edu.cn (Y.W.); limeng1232022@163.com (M.L.); 22s008118@stu.hit.edu.cn (X.L.); 2 State Key Laboratory for High Performance Tools, Zhengzhou Research Institute for Abrasives & Grinding Co., Ltd., Zhengzhou 450001, China; ljf19981996@163.com; 3 School of Mechanical Engineering, Donghua University, Shanghai 201620, China; wcjunm@dhu.edu.cn

**Keywords:** chemical–mechanical polishing, diamond, ReaxFF MD, oxidation reaction

## Abstract

Understanding the interfacial oxidation behavior during chemical–mechanical polishing (CMP) of diamond is important for controlling surface chemical modification and subsequent material removal. In this study, reactive force field molecular dynamics (ReaxFF MD) simulations were employed to comparatively investigate the atomistic evolution of interfacial oxidation, charge transfer, chemical species, and bond structures of single-crystal diamond in •OH- and H_2_O_2_-containing environments under different pressure conditions. The results show that oxidant chemistry and mechanical loading jointly regulate the kinetics and pathway selectivity of interfacial reactions. Elevated pressure promotes early-stage interfacial contact, surface activation, and oxidant consumption; however, the subsequent evolution of C–O bonds is also governed by oxidant-specific reaction kinetics and the availability of reactive species. In the •OH system, highly reactive radicals interact directly with mechanically activated carbon sites, whereas oxidation in the H_2_O_2_ system is additionally constrained by the stepwise decomposition of H_2_O_2_ and the generation of reactive intermediates, resulting in a comparatively moderated and decomposition-limited oxidation pathway. The interfacial reactions involve the formation and dynamic evolution of C–O, C–OH, and C–H structures, reflecting the competition between oxidation and hydrogenation. XPS measurements after H_2_O_2_-based CMP provide qualitative experimental evidence for the formation of oxygen-containing functional groups on the diamond surface, supporting the occurrence of interfacial oxidation. These findings provide atomistic insight into the coupled effects of oxidant chemistry and mechanical loading during diamond CMP and may assist in the rational design of oxidation-assisted polishing processes.

## 1. Introduction

Single-crystal diamond exhibits significant application potential in high-end optical windows, semiconductor devices [1,2,3], precision cutting tools [4], and quantum information technologies owing to its extremely high hardness [5], superior thermal conductivity, a wide bandgap, outstanding chemical stability, and broad spectral transparency ranging from the deep ultraviolet to the far infrared [6]. With the rapid advancement of third-generation semiconductor technologies, the demand for single-crystal diamond in high-frequency, high-voltage, and high-temperature electronic devices has become increasingly urgent [7,8]. Consequently, the requirements for its surface quality have evolved from mere macroscopic flatness to atomic-level flatness and a subsurface damage-free state [9,10,11]. However, its extreme hardness and chemical stability also render it a typical “difficult-to-machine material”, particularly in achieving nanoscale surface roughness and low-damage processing, which poses significant challenges [12,13]. Traditional mechanical polishing, which relies on the aggressive cutting action of diamond abrasives, is highly prone to introducing microcracks, residual stresses, and subsurface damage [14,15,16]. This not only reduces material utilization efficiency but also severely constrains device performance and reliability. Therefore, the development of efficient and damage-free precision polishing techniques has emerged as one of the critical bottlenecks in the application of diamond.

To achieve atomically smooth surfaces, it is essential to subject the material surface to high-precision and low-damage processing and control, thereby effectively eliminating surface defects and subsurface damage layers generated during the manufacturing process [17,18,19]. As a pivotal means of realizing ultra-smooth and high-quality diamond surfaces, polishing technology has consistently remained a focal point of research in this field. Early diamond polishing techniques primarily relied on thermochemical polishing (TCP) and mechanical polishing (MP). TCP is a processing technique based on thermodynamics and interfacial chemical reactions [20]. Material removal is primarily achieved through the diffusion of carbon atoms into a metal substrate at high temperatures, the structural transformation of diamond into graphite, and surface oxidation reactions. Ma et al. [21] employed a heated iron disk to polish a diamond specimen for two hours, successfully reducing the surface roughness from 9.6 μm to 16 nm. Although this method yields superior surface quality, it imposes stringent requirements on processing conditions. In contrast, MP relies primarily on the intense abrasive action of diamond particles to achieve surface planarization. Li et al. [22] utilized a water-based slurry to precision-polish polycrystalline diamond, achieving a surface roughness (Ra) of 96 nm at a material removal rate of 7.097 μm/h. However, the violent interactions between hard abrasives and the diamond surface are prone to generating scratches and embedding contaminants, making it difficult to meet the requirements for atomic-level flatness [23]. Overall, while traditional TCP and MP can achieve high material removal efficiency on a macroscopic scale, their reliance on intense mechanical forces or extreme thermal conditions makes it difficult to avoid introducing microcracks, residual stresses, and structural defects, thereby limiting further improvements in surface quality. To efficiently achieve atomically smooth surfaces, researchers have conducted extensive investigations into diamond polishing technologies. Currently, advanced polishing techniques mainly include ion beam polishing (IBP) [24], laser polishing (LP) [25], gas cluster ion beam polishing (GCIBP) [26,27], laser-assisted polishing (LAP) [28,29], ultraviolet-assisted polishing (UVAP) [30], and chemical–mechanical polishing (CMP) [31,32]. Mi et al. [33] utilized IBP to process diamond workpieces with localized defects. While this method effectively removed surface defects, its processing efficiency was relatively low, making it unsuitable for large-area applications. Liu et al. [34] employed a pulsed UV laser to polish CVD diamond, achieving a surface roughness (Ra) of 8.02 nm. Wang et al. [35] demonstrated a significant improvement in surface roughness from 334 nm to 0.5 nm using GCIBP. Furthermore, Liu et al. [36] proposed a LAP method that enabled uniform removal of diamond material with thicknesses of several micrometers. However, laser-processed surfaces often exhibit a sawtooth-like morphology and remain relatively rough. Meanwhile, Liu et al. [37] investigated the tribological behavior of single-crystal diamond using UVAP and confirmed that UV irradiation can accelerate the material removal rate by catalyzing interfacial reactions. Although these methods have improved diamond surface quality, achieving both high efficiency and atomic-level surface quality without introducing subsurface damage or non-diamond phase contamination remains a significant challenge. Chemical–mechanical polishing integrates chemical reactions with mechanical removal, and provides a promising solution by surface modification and efficient removal of reaction products [38,39]. This method has demonstrated outstanding performance in ultra-precision processing and is widely regarded as a key approach for achieving atomically smooth diamond surfaces.

Since the use of high-hardness abrasives during polishing inevitably introduces mechanical damage, achieving ultra-smooth and damage-free surfaces typically relies on a synergistic mechanism involving soft abrasives and chemical surface modification. In recent years, CMP has attracted considerable attention in precision diamond processing. Liao et al. [40] developed a novel SiO_2_ polishing slurry that achieved effective material removal by promoting oxidation reactions on the diamond surface, thereby yielding ultra-smooth surfaces with a minimal damage layer. Xin et al. [41] employed a CMP method utilizing CeO_2_-containing abrasives and achieved a material removal rate of 206.4 nm/h, together with a surface roughness (Ra) of 0.6 nm and a plastic damage layer thickness of 3.242 nm. Based on a hydroxy-oxidation mechanism, Yuan et al. [42] proposed an atomic-scale planarization approach that suppresses the influence of grain orientation and grain boundaries on surface quality during the CMP of polycrystalline diamond, achieving a surface roughness of 0.366 nm. Yu et al. [43] reported a green and efficient CMP process based on rapid surface oxidation, achieving a material removal rate of 1168 nm/h, a surface roughness (Sa) of 0.079 nm, and a subsurface damage layer thickness of 0.66 nm. Wang et al. [44] conducted CMP on single-crystal diamond using a polishing slurry containing Fenton reagents, elucidating the synergistic roles of chemical oxidation and mechanical removal, and achieving a surface roughness of 0.856 nm. Zhu et al. [45] further demonstrated that optimizing CMP parameters enables a material removal rate of 109.6 nm/min and a surface roughness of 0.6 nm, attributing the enhanced efficiency to surface oxidation reactions and the formation of a softened amorphous layer. Although numerous experimental studies have demonstrated that efficient and low-damage diamond processing can be achieved through the synergistic interplay of surface chemical modification and CMP, traditional experimental methods are difficult to capture the bond state evolution and interfacial reaction mechanisms at the atomic scale. In contrast, molecular dynamics (MD) simulations, especially those based on reactive force field (ReaxFF), provide an effective way to elucidate interfacial chemical reactions and their coupling mechanisms with mechanical interactions [46,47]. Shi et al. [48] employed ReaxFF MD simulations to investigate diamond CMP in hydroxyl-rich environments, identifying the formation of C–C and C–O bonds at the interface as critical prerequisites for subsequent material removal under sliding. Guo et al. [49] simulated CMP using SiO_2_ abrasives and revealed the dual role of surface oxidation in carbon atom removal, demonstrating that material removal arises from the synergy between chemical reactions and mechanical actions. Yuan et al. [50] further examined the effects of pressure and sliding velocity, showing that increased pressure enhances C–C bond reconstruction and fracture during carbon atom removal. In addition, Zhang et al. [51] investigated ultraviolet (UV)-assisted CMP, demonstrating that UV irradiation can enhance the material removal rate while reducing surface roughness. Liu et al. [52] explored plasma-assisted polishing via ReaxFF MD simulations, revealing that oxygen plasma promotes the formation of C–O bonds, thereby leading to surface graphitization and amorphization. Although previous ReaxFF studies have separately revealed OH-assisted interfacial bond formation, oxidation-assisted carbon removal in H_2_O_2_/H_2_O environments, and pressure- and velocity-dependent interfacial behaviors. However, a direct comparison of how oxidant chemistry modifies the pressure-dependent reaction kinetics and pathway selectivity under otherwise identical interfacial conditions remains limited. Moreover, the atomic-scale mechanism by which polishing pressure modulates reaction pathway selectivity (i.e., oxidation vs. hydrogenation) and oxidation kinetics (controlled vs. aggressive) remains unclear.

Therefore, the present study focuses on the coupled effects of oxidant type and mechanical pressure by jointly analyzing reactant consumption, charge redistribution, and the dynamic evolution of C–O and C–H bonds. This study makes the following contributions: (i) a direct comparative analysis of •OH and H_2_O_2_ systems under identical simulation conditions, revealing that H_2_O_2_ promotes controlled surface modification rather than aggressive oxidation; (ii) a quantitative demonstration that pressure not only accelerates oxidation but also qualitatively alters bond evolution dynamics (C-O vs. C-H competition); and (iii) an integrated simulation–experiment validation using XPS under H_2_O_2_ conditions, which confirms the predicted formation of oxygen-containing functional groups. The study elucidates the underlying oxidation mechanisms at the atomic scale, providing a theoretical foundation for achieving highly efficient, low-damage, and atomically smooth surfaces. Specifically, this work focuses on the evolution of surface oxidation reactions, interfacial charge transfer, variations in chemical composition, and the dynamic changes in bond structures, thereby clarifying the coupling between oxidation reactions and mechanical loading. These findings are expected to provide theoretical support for optimizing diamond CMP processes and surface modification techniques, and to facilitate the advancement of wide-bandgap semiconductor materials toward atomic-scale precision manufacturing.

## 2. Methods and Conditions

### 2.1. ReaxFF MD Simulations

Diamond exhibits exceptional properties, rendering it a promising material for a wide range of advanced applications. However, its extreme hardness and strong chemical inertness pose significant challenges to achieving damage-free and ultra-smooth surfaces [53,54,55]. CMP has emerged as an effective approach for atomic-scale planarization of diamond, in which material removal is governed by the synergistic interplay between surface chemical reactions and mechanical actions [56,57]. In particular, surface oxidation reactions play a decisive role in governing the material removal mechanisms and enhancing surface integrity. Therefore, elucidating the oxidation behavior of diamond surfaces during CMP is critical for understanding the underlying removal mechanisms and their impact on surface integrity.

Given that the CMP process involves significant interfacial chemical reactions, traditional potential functions struggle to accurately describe the behaviors of chemical bond breaking and reconstruction. Consequently, this study employs the reactive force field to conduct MD simulations investigating the oxidation reaction behaviors during diamond CMP under various polishing slurries and pressures [58,59]. The work utilizes a single-crystal diamond (100) plane as the substrate material, SiO_2_ as the abrasive, and •OH and H_2_O_2_ as the oxidizing agents within the polishing slurry. As illustrated in Figure 1, the MD model adopts a typical sandwich structure consisting of an SiO_2_ abrasive layer (256 Si atoms and 448 O atoms), a polishing slurry layer, and a diamond substrate (1536 C atoms), thereby constructing an MD simulation system with dimensions of approximately 28.4 × 28.4 × 45.4 Å^3^. The SiO_2_ abrasive was modeled as a local nanoscale surface patch rather than as an entire abrasive particle. Because the simulated lateral dimension is much smaller than the 50 nm experimental particle diameter, the local surface of the experimental particle is approximated as planar. Accordingly, the present model is intended to examine local interfacial reaction mechanisms rather than the complete contact mechanics of an individual abrasive particle. The diamond substrate was constructed by cleaving an ideal bulk diamond crystal along the (100) plane. The exposed surface was initially unpassivated, with no H- or O-termination, predefined reconstruction, vacancies, steps, or other artificial defects introduced. The model was subsequently subjected to energy minimization and thermal equilibration using the ReaxFF potential before compression and sliding. Therefore, the initial model represents an idealized reactive diamond (100) surface rather than a chemically terminated CVD surface. As shown in Figure 1a, a diamond crystal approximately 10 Å thick is placed at the bottom of the system, while an SiO_2_ crystal with a thickness of 12 Å is positioned at the top. The intervening space is filled with a solution containing 100 H_2_O molecules and 100 hydroxyl radicals (•OH), resulting in a total of 2740 atoms in the simulation system. As shown in Figure 1b, a similar configuration is employed, except that the solution consists of 100 H_2_O molecules and 100 H_2_O_2_ molecules, yielding a total of 2940 atoms. The atoms within the bottom 3 Å of the diamond substrate are defined as a fixed layer, whereas those within the topmost 3 Å of the SiO_2_ abrasive are assigned as a rigid moving layer. Periodic boundary conditions are applied in the x and y directions to represent an extended diamond surface, while a free boundary condition is imposed along the z direction. The detailed simulation parameters are summarized in Table 1. Overall, the model consists of a rigid moving layer, a free layer of SiO_2_ abrasive, a polishing slurry layer, a free layer of the diamond substrate, and a fixed layer. Atoms within the free layers are allowed to move freely under interatomic interactions, whereas atoms in the fixed layer are constrained to simulate the diamond workpiece anchored to the polishing pad. To prevent atomic escape or loss along the z direction [60,61,62], reflecting walls are introduced at both the top and bottom boundaries of the simulation cell in the z direction.

Prior to the simulation, the system was first subjected to energy minimization to eliminate unfavorable atomic contacts, with energy and force convergence thresholds of 1.0 × 10^−4^ kcal/mol and 1.0 × 10^−4^ kcal/(mol·Å), respectively. The initial system was then equilibrated in the canonical (NVT) ensemble using a Langevin thermostat at 300 K. During equilibration, normal pressures of 10 MPa, 50 MPa, 100 MPa, and 150 MPa were applied along the z direction to achieve a stable configuration and appropriate density of the polishing slurry. It should be noted that the pressures applied in the MD model represent nanoscale interfacial loading conditions and are not intended to correspond directly to the nominal pressure applied to the macroscopic CMP specimen. Following equilibration in the NVT ensemble, the MD simulations were conducted under the microcanonical (NVE) ensemble to investigate the intrinsic dynamical behavior of the system while conserving the total energy. All atoms in the mobile regions were allowed to move freely according to interatomic forces, and the system was monitored over a predetermined simulation time to ensure equilibration and reliability of the results [63,64,65]. The entire simulation procedure was divided into three sequential stages: (1) The SiO_2_ abrasive was gradually pressed toward the diamond substrate along the z direction, compressing the polishing slurry and raising the system pressure to the target value. This process lasts for 10 ps. (2) The system was equilibrated under the target pressure for 50 ps to achieve structural stability and full contact between the polishing slurry and the diamond substrate surface. (3) The SiO_2_ abrasive was driven to slide along the x direction at a constant velocity of 10 m/s for 150 ps, allowing investigation of the oxidation behavior of diamond surfaces during CMP. All ReaxFF MD simulations were conducted using a time step of 0.1 fs, and the Velocity-Verlet algorithm was utilized to integrate Newton’s equations of motion. In this study, the Si/C/O/H force field within the ReaxFF framework was employed to describe the interactions among C, H, O, and Si atoms [66]. All simulations were carried out using the large-scale atomic/molecular massively parallel simulator (LAMMPS) software, and the simulation results were visualized and post-processed using the Open Visualization Tool (OVITO). Electrostatic interactions were treated using the charge equilibration (QEq) method [67,68,69], which updated atomic charges at every time step. Throughout the simulations, the evolution of bonding states, formation of reaction products, and charge redistribution within the system were systematically tracked to elucidate the mechanisms underlying surface oxidation during the diamond CMP process. Chemical species and bond populations were identified based on the bond-order information generated by the ReaxFF potential in LAMMPS. The standard ReaxFF species-analysis procedure was employed, in which two atoms were considered bonded when their bond order exceeded 0.30. The same bond-order cutoff was applied to all atomic pairs, including C–O, C–H, O–H, and O–O, without condition-dependent adjustment. Molecular species were determined from the connectivity of bonded atoms and their elemental composition. Bond-order information was sampled every 1000 timesteps, and species information was output every 1000 timesteps. No additional minimum lifetime filter was imposed. OVITO was used for visualization of atomic configurations rather than for defining chemical bonds or molecular species.

### 2.2. Experimental Setup

To validate the reliability of the ReaxFF MD simulation results, this study conducted CMP experiments on diamond substrates using a TriboLab CMP automatic polishing machine (Bruker, CA, USA). The diamond substrates used in the experiments were single-crystal diamonds prepared via chemical vapor deposition (CVD) by the Zhengzhou Research Institute for Abrasives & Grinding Co., Ltd. (Zhengzhou, China), with dimensions of 10 mm × 10 mm × 1 mm. To ensure uniform dispersion of the abrasive particles and suppress agglomeration during the polishing process, the polishing slurry was continuously delivered to the surface of the polishing pad at a constant flow rate using a magnetic stirrer and a peristaltic pump system, thereby ensuring the stability of the polishing process. The polishing parameters were set as follows: a polishing load of 30 N and a slurry flow rate of 15 mL/min. The rotation speed of the polishing disk was 80 r/min, and the rotation speed of the upper holder was 60 r/min. In addition, the upper holder executed a reciprocating radial motion across the polishing disk at a frequency of 3 cycles per minute, and the total polishing duration was 1 h. The detailed polishing parameters are listed in Table 2. The polishing slurry was prepared using SiO_2_ abrasive particles with an average diameter of 50 nm and a 30 wt% hydrogen peroxide (H_2_O_2_) solution, mixed at a mass ratio of 1:3. Following the polishing process, X-ray photoelectron spectroscopy (XPS, Thermo Fisher, Waltham, MA, USA) was employed to characterize the chemical bonding states on the diamond surface. The diamond surface was characterized by XPS using a Thermo Scientific K-Alpha system (Thermo Fisher, Waltham, MA, USA) equipped with a monochromatic Al Kα X-ray source (1486.6 eV). During the measurements, the anode voltage and emission current were set to 12 kV and 6 mA, respectively. By systematically comparing the XPS spectra, the oxidation behavior of carbon atoms on the diamond surface was analyzed, thereby providing qualitative experimental evidence for the occurrence of surface oxidation predicted by the ReaxFF MD simulations and providing theoretical foundations for the subsequent optimization of diamond CMP processes.

## 3. Results and Discussions

During chemical–mechanical polishing, material removal from diamond is fundamentally driven by the synergistic interplay between interfacial friction and chemical reactions. The polishing slurry chemistry and the applied load are considered key factors in determining the reaction pathways and removal efficiency. In oxidative polishing systems, distinct reaction mechanisms arise from different active species, such as hydroxyl radicals (•OH) and hydrogen peroxide (H_2_O_2_), leading to markedly different oxidation behaviors at the diamond surface [70]. Concurrently, the applied polishing pressure exerts a substantial influence on interfacial reaction kinetics by modulating the contact conditions and stress distribution between the abrasive particles and the diamond surface. Consequently, the coupled effects between the slurry chemistry and the mechanical load collectively dictate the interfacial bonding evolution and surface oxidation processes during diamond CMP. In this section, ReaxFF MD simulations are performed to systematically investigate the evolution of diamond surface oxidation under varying polishing pressures in both •OH and H_2_O_2_ environments. This study aims to elucidate the atomic-scale mechanisms underlying chemical–mechanical synergy, thereby providing a theoretical insight for the optimization of diamond CMP processes.

### 3.1. Analysis of Simulation Results in •OH Environment

In oxidative CMP systems, •OH radicals are widely recognized as highly reactive species that can alter the chemical properties of diamond surfaces. Owing to their strong oxidative capability, •OH radicals can readily attack surface C–C bonds of diamond, inducing bond cleavage and facilitating the formation of oxygen-containing functional groups. This process is expected to weaken the diamond lattice at the interface and promote the oxidation of carbon atoms on the diamond surface under applied pressure. Therefore, this section presents a systematic analysis of ReaxFF MD simulation results obtained under •OH environments at 10 MPa and 100 MPa, which is crucial for elucidating the evolution of bond reconstruction and surface oxidation behavior.

Figure 2 illustrates the atomic-scale evolution of diamond surface oxidation under an applied pressure of 10 MPa in an •OH environment. At the initial stage of loading, SiO_2_ abrasive particles progressively approach the diamond surface, leading to gradual compression of the polishing slurry and the establishment of incipient interfacial contact. This early contact triggers the onset of interfacial reactions and induces subtle structural perturbations at the outermost atomic layers of the diamond surface. Owing to the relatively low external load at this stage, the real contact area remains limited, and a fully developed, intimate three-phase contact among the abrasive particles, slurry medium, and diamond substrate has not yet been achieved. Consequently, the adsorption and reaction of •OH radicals are spatially restricted to a few localized high-energy sites, such as undercoordinated carbon atoms or surface defects, resulting in the formation of a limited number of C–O and C–OH structures and only minor perturbations to the surface structure. As the applied pressure gradually increases, the interfacial gap between the SiO_2_ abrasives and the diamond substrate decreases, accompanied by further densification of the slurry confined within the contact region. This compression effect enhances the effective concentration and accessibility of reactive •OH radicals near the interface, thereby promoting more frequent interactions with surface carbon atoms. As a result, the population of oxidized carbon atoms on the surface increases progressively. However, even when the pressure reaches the preset value of 10 MPa, the interfacial gap decreases and the slurry is further compressed, enhancing the interaction probability between •OH radicals and surface carbon atoms. Consequently, the number of oxidized carbon species gradually increases. However, the overall oxidation extent remains relatively mild, and the induced chemical modifications are largely confined to the surface-layer atoms and are insufficient to cause pronounced lattice distortion or structural perturbation of the diamond crystal. Upon entering the equilibration stage, the interfacial contact state tends toward stability. Nevertheless, the contact stress and stress concentration remain insufficient to continuously activate the subsurface carbon atoms, thereby hindering the progression of interfacial chemical reactions. As a result, oxidation reactions are largely confined to localized regions of the surface layer. In addition to oxygen-containing functional groups, a small number of C–H bonds are observed. The formation of these C–H structures can be attributed to the passivation of mechanically activated carbon atoms through interactions with hydrogen species derived from the aqueous environment. This hydrogen termination effectively stabilizes dangling bonds and reduces the overall chemical reactivity of the surface [71]. During this stage, the diamond lattice largely retains its structural integrity, indicating that the strong oxidative potential of •OH radicals is not fully manifested under low-pressure conditions. In the subsequent sliding stage, interfacial shear forces begin to contribute to the reaction process. However, under the relatively low pressure of 10 MPa, the magnitude of shear stress remains insufficient to induce significant bond reconstruction or large-scale structural disruption at the surface. Although the sliding motion enhances the dynamic contact between •OH radicals and reactive surface sites, insufficient mechanical activation restricts the progression of the surface oxidation reaction.

Figure 3 presents the atomic-scale evolution of diamond surface oxidation under an applied pressure of 100 MPa in an •OH environment. During the high-pressure loading stage, SiO_2_ abrasive particles rapidly establish intimate contact with the diamond surface, leading to a substantial expansion of the real contact area. Under such conditions, the bonding states of surface carbon atoms are strongly perturbed by the elevated normal stress, rendering C–C bonds more susceptible to activation. Concurrently, the increased frequency and intensity of interfacial contact events enhance the probability of interactions between •OH radicals and reactive carbon sites. As a consequence, the formation of oxygen-containing functional groups, particularly C–O bonds, is markedly promoted even at the early stage of loading, indicating that oxidation is effectively activated from the outset. As the system transitions into the equilibration stage, the persistent stress field sustained by the high pressure further intensifies the kinetics of the interfacial reaction. Under the synergistic coupling of mechanical stress and chemical reactivity, surface carbon atoms undergo continuous bond reconstruction, leading to pronounced oxidation and structural distortions within the near-surface region. During the sliding stage, the contribution of shear stress becomes increasingly significant. The combination of high pressure and substantial shear forces promotes the dynamic renewal of reactive sites, effectively exposing fresh, undercoordinated carbon atoms that readily interact with •OH radicals, thereby sustaining and accelerating the oxidation reaction [72]. Compared with the low-pressure condition, the mechanochemical coupling under 100 MPa is enhanced, resulting in a higher oxidation rate and a greater extent of surface modification. This intensified oxidation behavior can be attributed to several factors. First, elevated pressure increases interfacial contact and promotes the formation of mechanically activated surface sites, thereby increasing the probability of reactions between reactive oxygen species and surface carbon atoms. Second, the enhanced confinement of the polishing slurry increases the local availability and effective concentration of reactive •OH species at the interface [73]. Third, the combined normal and shear stresses facilitate continuous atomic-scale restructuring, thereby sustaining the reactivity of the surface. In contrast, under low-pressure conditions, insufficient stress levels limit both bond activation and reactive-species accessibility, resulting in sluggish oxidation kinetics and minimal structural perturbation. In summary, polishing pressure plays a role in governing the oxidation behavior of diamond in an •OH environment. Low-pressure conditions are characterized by limited mechanochemical activation and slow oxidation kinetics, whereas high-pressure conditions enhance interfacial reactivity, promote bond activation, and accelerate oxidation-induced structural evolution. These findings highlight the critical role of mechanical loading in modulating the interplay between stress, chemical reactions, and structural transformation during the CMP process.

During the diamond CMP process, interactions between surface carbon atoms and oxidizing species are accompanied by significant charge transfer and redistribution [74]. Accordingly, analyzing the evolution of surface charge provides critical insight for understanding the underlying reaction mechanisms. Figure 4 shows the charge distribution and its temporal evolution on the diamond surface obtained by the charge equilibration (QEq) method in an •OH environment using ReaxFF MD simulations. Under a pressure of 10 MPa, the overall charge distribution on the diamond surface remains relatively uniform, with only slight charge transfer occurring in a few localized regions. During the initial loading stage, adsorption reactions involving high-energy carbon atoms induce electron transfer, leading to a slight positive charge on surface carbon atoms. However, owing to the limited interfacial contact, such charge redistribution is primarily confined to scattered regions. As the system enters the equilibration stage, the charge distribution gradually stabilizes, and the charge transfer process is characterized by gradual accumulation. The formation of a small number of C–O bonds, accompanied by continuous yet limited electron transfer, slightly increases the positive charge on carbon atoms. During the sliding stage, interfacial shear enhances the contact frequency between •OH radicals and surface active sites, thereby further promoting charge transfer. In contrast, under a pressure of 100 MPa, the charge distribution on the diamond surface becomes more pronounced. During the initial loading stage, a large number of surface carbon atoms undergo electron transfer, resulting in pronounced positive charge accumulation. In the equilibration stage, the high-pressure environment sustains a high level of reactivity, further enhancing charge transfer and enabling its expansion over a broader surface area. With the continuous formation of C–O bonds, contiguous charge-enriched regions emerge on the surface, indicating that the diamond lattice is undergoing a transition from an ordered to a more disordered structure. Upon entering the sliding stage, interfacial shear and charge transfer exhibit a significant synergistic effect. On the one hand, shear stress continuously exposes new active sites, facilitating additional charge transfer. On the other hand, pre-existing charge-enriched regions reduce the reaction barrier, thereby accelerating oxidation reactions. Overall, the results demonstrate that charge transfer occurs predominantly on surface carbon atoms in the •OH environment. The polishing pressure plays a critical role in governing the spatial distribution and temporal evolution of surface charge by modulating the interfacial contact state and stress distribution. Under high-pressure conditions, enhanced charge transfer reflects a higher degree of surface oxidation.

In the •OH oxidation system, H_2_O molecules and •OH radicals serve as both primary reactants and key intermediates, and their population changes directly reflect the extent of oxidation and the dynamic equilibration state of the system [75]. To elucidate the influence of pressure on the oxidation behavior of the diamond surface, the temporal evolution of H_2_O molecules, •OH radicals, and the total number of molecules and species was systematically analyzed under different pressure conditions in an •OH environment, as shown in Figure 5. During the initial stage (0–10 ps), the populations of H_2_O, •OH, total molecules, and species all exhibit a rapid decline. This decrease is more pronounced under 100 MPa than under 10 MPa, indicating that elevated pressure accelerates the consumption of reactive species. Under high-pressure conditions, the enhanced interfacial contact and increased collision frequency promote rapid involvement of slurry constituents in chemical reactions, leading to intensified interactions with the diamond surface and among slurry species. In contrast, under low-pressure conditions, the reduction in species populations is more gradual, and the system stabilizes at higher levels, suggesting a lower overall reaction rate. During the subsequent equilibration (10–60 ps) and sliding (60–150 ps) stages, the populations of different species exhibit dynamic fluctuations under both pressure conditions. At 10 MPa, the number of H_2_O molecules and the total number of species remain relatively high, indicating that the reaction system is weakly activated and dominated by limited consumption of reactants. However, under 100 MPa, the number of •OH radicals and the total number of species display fluctuations with greater amplitude and frequency. This behavior reflects the highly dynamic nature of the reaction system under high-pressure conditions. The pronounced fluctuations observed at 100 MPa can be attributed to the enhanced mechanochemical coupling at the interface. Quantitative contact analysis shows that increasing the applied pressure increases the number of interfacial contact atoms and decreases the mean interfacial separation. These changes are accompanied by an earlier increase in the oxidized-carbon fraction and C–O bond population, indicating that enhanced interfacial contact facilitates mechanochemical activation and promotes the initiation of oxidation reactions. In contrast, under low-pressure conditions, both the generation and consumption rates of reactive species remain limited due to insufficient activation of interfacial reactions. Consequently, the system evolves more steadily, with smaller fluctuations and a slower overall reaction rate. These results demonstrate that pressure plays a critical role in regulating the dynamic balance between species generation and consumption, thereby governing the kinetics and extent of oxidation in the •OH system.

The evolution of C–O and C–H structures provides indirect but meaningful evidence for the degree of interfacial oxidation and variations in surface reactivity during diamond CMP. Specifically, the population of C–O bonds reflects the initiation and progression of oxidation reactions, whereas the evolution of C–H bonds is closely associated with hydrogen-induced surface passivation and competing reaction pathways. Figure 6 presents the temporal evolution of C–O and C–H bond populations during CMP in an •OH environment. The formation of C–O bonds exhibits pronounced pressure dependence and distinct staged characteristics. Under 10 MPa, the population of C–O bonds remains low throughout the simulation period. During the initial stage (0–10 ps), negligible C–O bond formation is observed, indicating that interfacial reactions are not effectively activated under low-pressure conditions. In the subsequent equilibration stage (10–60 ps), the C–O population increases only gradually with minor fluctuations, suggesting that oxidation is constrained by limited interfacial contact and insufficient activation of surface carbon atoms. During the sliding stage (60–150 ps), although shear-induced interactions promote a slight increase in C–O bond formation, the overall population remains low and exhibits pronounced fluctuations, reflecting the localized and intermittent nature of oxidation under low pressure. In contrast, the population of C–O bonds at 150 MPa increases rapidly during the initial stage and reaches a relatively high level in the equilibration stage, indicating that elevated pressure enhances interfacial contact and facilitates rapid reaction initiation. During the sliding stage, although fluctuations persist due to dynamic bond breaking and reformation, the C–O population remains high and continues to increase gradually. This behavior demonstrates that high pressure not only promotes the onset of oxidation but also sustains its progression by continuously activating surface sites. The evolution of C–H bonds differs markedly from that of C–O bonds, highlighting the competitive nature of hydrogenation and oxidation pathways. At 10 MPa, C–H bonds are nearly absent during the initial stage and appear only in small quantities during the equilibration stage, followed by a significant increase accompanied by large fluctuations during the sliding stage. This behavior suggests that insufficient oxidation under low-pressure conditions leaves a portion of active sites on the surface available for hydrogen termination, leading to the formation of C–H bonds. The pronounced fluctuations further indicate that these bonds are relatively unstable and undergo continuous formation and rupture under shear stress. Under 100 MPa conditions, the population of C–H bonds remains consistently low with relatively minor fluctuations throughout the simulation. Although a slight increase is observed during the sliding stage, the overall level remains lower than that observed under low-pressure conditions. This indicates that surface carbon atoms preferentially react with oxygen species in a high-pressure •OH environment, thereby suppressing hydrogenation reactions. Overall, these results demonstrate that polishing pressure plays a critical role in regulating species evolution and reaction pathways in an •OH environment. By modulating interfacial contact conditions, stress levels, and reaction kinetics, pressure governs the balance between oxidation and hydrogenation processes, ultimately determining the extent and nature of surface modification during diamond CMP.

### 3.2. Analysis of Simulation Results in H_2_O_2_ Environment

H_2_O_2_ is widely employed as a representative oxidizing agent due to its moderate reactivity and controllable decomposition behavior. Unlike highly reactive species such as •OH radicals, H_2_O_2_ typically undergoes stepwise decomposition to generate reactive intermediates, resulting in a more gradual and regulated oxidation process at the diamond surface. This relatively mild oxidation pathway promotes the formation of oxygen-containing functional groups without causing excessive lattice disruption [76]. In this section, the oxidation behavior and structural evolution of diamond under H_2_O_2_ environment at 10 MPa and 100 MPa are systematically investigated to provide insights into the evolution of surface oxidation and bond reconstruction during CMP.

Figure 7 depicts the atomic-scale evolution of diamond oxidation on the surface under 10 MPa in an H_2_O_2_ environment. During the initial stage of compression, the system largely retains its pristine state, with a well-defined spatial distribution of the diamond substrate, SiO_2_ abrasive particles, and polishing slurry. As external pressure is applied, the SiO_2_ abrasives progressively approach the diamond surface, bringing a small amount of polishing slurry into contact with the interfacial region. Constrained by the relatively low applied pressure, both the interfacial contact area and contact strength remain limited, resulting in a rather dispersed distribution of the polishing slurry within the interface. Under these conditions, H_2_O_2_ molecules are primarily adsorbed onto the diamond surface through physisorption or weak chemisorption. The decomposition of H_2_O_2_ has not yet been activated, and only a limited amount of reactive species is generated, leading to the formation of a sparse distribution of oxygen-containing functional groups, predominantly C–OH structures. As the applied pressure increases, the polishing slurry undergoes further compression and densification, increasing the probability of molecular interactions at the interface. This triggers the onset of oxidation reactions. However, due to the insufficient activation of surface carbon atoms under relatively low stress conditions, the majority of C–C bonds remain structurally stable, with only minor perturbations occurring at isolated reactive sites. As a result, the overall extent of oxidation remains negligible. When the pressure reaches the predetermined level of 10 MPa, chemisorption processes at the interface become more pronounced. Reactive species generated from H_2_O_2_ decomposition begin to interact more frequently with surface carbon atoms, inducing the surface-layer carbon atoms to exhibit discernible changes. Despite this, the oxidation process is still kinetically constrained, and the transformation of carbon atoms into oxygen-containing structures proceeds at only a relatively slow rate. Upon entering the equilibration stage, the interfacial contact state stabilizes. However, under the 10 MPa condition, the stress level remains insufficient to catalyze the decomposition of H_2_O_2_ or to substantially enhance the kinetics of the interfacial reactions. Therefore, the oxidation process manifests as a slow and continuous surface functionalization phenomenon. The density of C–O bonds gradually increases, and a portion of surface carbon atoms is transformed into oxygen-containing groups, such as C–OH and C–O–C structures. However, the overall oxygen incorporation remains limited, and the diamond lattice largely preserves its structural integrity. No evident lattice distortion or structural degradation is observed, indicating that this stage is dominated by mild oxidation and surface passivation rather than by aggressive bond-breaking events. During the subsequent sliding stage, the introduction of interfacial shear stress promotes the dynamic renewal of contact regions and enhances the transport and exchange of reactants within the confined slurry layer. This process increases the frequency of interactions between H_2_O_2_ (as well as its decomposition products) and reactive surface sites, thereby facilitating a more uniform distribution of oxidation across the surface. Nevertheless, under a pressure of 10 MPa, the magnitude of shear stress remains insufficient to induce significant mechanochemical activation. Consequently, its direct catalytic effect on the surface oxidation reaction remains limited. The observed behavior can be fundamentally attributed to the intrinsic reaction pathway of H_2_O_2_, which requires stepwise decomposition to generate highly reactive radical species (such as •OH and HO_2_•). Under low-pressure conditions, both the decomposition kinetics of H_2_O_2_ and the effectiveness of interfacial contact are constrained, thereby limiting the overall reaction rate. Therefore, the process is characterized by a low reaction rate and minimal structural perturbation of the diamond surface.

Figure 8 displays the atomic-scale evolution of diamond oxidation on the surface under 100 MPa in an H_2_O_2_ environment. During the initial loading stage, the polishing slurry is rapidly compressed against the diamond surface, resulting in the formation of an intimate interfacial contact and a substantial increase in the contact area. Under these high-pressure conditions, the activation of surface carbon atoms is enhanced, while the decomposition of H_2_O_2_ is simultaneously promoted, leading to an increased generation of reactive oxygen species. At this stage, C–C bonds at the surface become more susceptible to activation, and the formation of oxygen-containing functional groups (such as C–O bonds) is markedly intensified compared to low-pressure conditions. Despite this enhancement, the oxidation process proceeds in a progressive and controlled manner rather than through abrupt or oxidation events. During the equilibration stage, the sustained high load maintains continuous contact stress and preserves a chemically active interfacial environment, thereby promoting the further progression of oxidation reactions. Under the coupled effects of pressure and chemical interactions, surface carbon atoms gradually undergo bond reconstruction, leading to the steady evolution of surface oxidation. Nevertheless, in contrast to the •OH environment, the oxidation behavior in the H_2_O_2_ environment remains kinetically moderated and spatially selective. Oxidation preferentially occurs at high-energy sites rather than propagating uniformly across the lattice. Although localized lattice distortions and bond rearrangements are observed, the overall diamond structure retains a relatively high degree of crystallographic integrity, indicating the absence of large-scale structural degradation. During the sliding stage, the interplay between elevated shear stress and the continuous supply of oxidizing species further accelerates the interfacial reaction process. The sliding motion facilitates the continuous exposure of fresh surface sites, thereby increasing the oxidation rate. However, due to the intrinsically limited concentration and reactivity of active species generated from H_2_O_2_ decomposition, the mechanochemical activation induced by shear stress remains insufficient to drive extensive subsurface oxidation or deep bond cleavage. Nonetheless, the overall extent of oxidation remains higher than that observed in low-pressure oxidation systems. Overall, the oxidation behavior of the diamond surface in an H_2_O_2_ environment is primarily governed by the interplay between oxidant decomposition kinetics and interfacial contact conditions. The stepwise decomposition of H_2_O_2_ into reactive radicals imposes an inherent kinetic limitation on the reaction rate, resulting in a process that is comparatively gradual and controllable. Increasing the polishing pressure enhances oxidation by improving interfacial contact conditions and accelerating the generation of reactive species, but the overall reaction behavior remains distinct from the rapid and oxidation observed in an •OH environments. This indicates that the oxidation rate of H_2_O_2_ is enhanced under high pressure, enabling surface carbon atoms to react more rapidly and thereby demonstrating the catalytic role of pressure in facilitating the reaction. Concurrently, these findings suggest that the H_2_O_2_ system is better suited for achieving the controllable oxidation of surface structures rather than for driving highly efficient, oxidation processes.

Figure 9 depicts the charge distribution and its temporal evolution on the diamond surface after oxidation in an H_2_O_2_ environment using ReaxFF MD simulations. Under a pressure of 10 MPa, the diamond surface exhibits a relatively uniform charge distribution, with only slight charge transfer occurring in localized areas. During the initial compression stage, H_2_O_2_ molecules predominantly interact with the surface via physical adsorption or weak chemisorption, resulting in limited electron transfer. Only a small fraction of reactive oxygen species generated from H_2_O_2_ decomposition participates in interfacial reactions, inducing minor electron transfer from surface carbon atoms and leading to localized accumulation of positive charge. As the system transitions into the equilibration stage, the charge distribution gradually tends to stabilize. Owing to the limited decomposition rate of H_2_O_2_ molecules and the limited availability of reactive species, the extent of charge transfer remains restricted, and no continuous or large-scale charge transfer behavior is observed. During the sliding stage, interfacial shear enhances reactant exchange and local contact. However, due to insufficient mechanical activation, the overall charge transfer is only moderately increased, maintaining a low level of surface charge polarization. Under a pressure of 100 MPa, the interfacial contact state is improved, and the driving force for the reaction is correspondingly enhanced, resulting in more pronounced charge redistribution. During the compression stage, the rapid establishment of intimate contact and the presence of stress concentration facilitate the decomposition of H_2_O_2_ molecules, thereby increasing the generation of reactive oxygen species. This leads to enhanced interactions between oxygen species and surface carbon atoms, accompanied by a noticeable increase in charge transfer, manifested as localized enrichment of positive charge on carbon atoms. In the steady-pressure stage, the sustained high-stress environment maintains an active interfacial reaction condition, promoting the expansion of charge transfer to a broader surface region. As a result, localized charge accumulation becomes more evident, indicating an increased degree of surface polarization and chemical reactivity. Upon entering the sliding stage, the combined effects of shear stress and ongoing reactions further drive the dynamic redistribution of charge. Nevertheless, due to the intrinsically limited concentration and reactivity of active species in the H_2_O_2_ system, large-scale and continuous charge transfer does not occur under a pressure of 100 MPa. This suggests that the oxidation process remains incomplete, although the extent of charge redistribution and associated surface activation is enhanced compared to that in an •OH environment under the same pressure.

To further reveal the kinetic characteristics of interfacial reactions in the H_2_O_2_ system, the temporal evolution of key species was analyzed from the perspective of reactant population. Figure 10 presents the evolution of H_2_O, H_2_O_2_, and the total number of molecules and species in the slurry system under different pressure conditions. As shown in Figure 10a, the number of H_2_O molecules decreases rapidly in the initial stage and then tends to stabilize, exhibiting certain differences under different pressure conditions. Under 10 MPa, the number of H_2_O molecules decreases to approximately 75 in the initial stage (0–10 ps), followed by a further decrease to about 60 during the equilibration stage (10–60 ps), after which it stabilizes with slight fluctuations. This indicates that significant reactions occur in the initial stage, with some H_2_O molecules participating in the reactions or transforming into other species, after which the system enters a relatively stable state. Under 100 MPa, the number of H_2_O molecules decreases more rapidly and tends to stabilize, but is generally lower than under 10 MPa conditions. This indicates that H_2_O molecules are more likely to participate in the reaction or be consumed under high-pressure conditions, reflecting an enhanced interfacial reactivity. As shown in Figure 10b, H_2_O_2_ molecules decompose rapidly in the initial stage and gradually approach zero in subsequent stages, which is an important indicator of the progress of the reaction. Under 10 MPa, the number of H_2_O_2_ molecules decreases from the initial value to below approximately 50 and then continues to decline slowly during the equilibration stage, eventually being nearly completely consumed in the sliding stage. This suggests that although the decomposition process of H_2_O_2_ is relatively slow under low pressure, it continues and is an important source of reactive species. Under 100 MPa, H_2_O_2_ molecules are rapidly consumed within a short time and are nearly depleted during the equilibration stage, demonstrating that high pressure promotes H_2_O_2_ decomposition, enhances the generation of reactive species, and accelerates the reaction kinetics of the system. As shown in Figure 10c, the total number of molecules in the system varies in the initial stage and then enters a stable fluctuation state. However, the total number of molecules decreases faster under high-pressure conditions and by a greater magnitude, indicating that elevated pressure promotes the molecular decomposition process and increases the number of small molecules or free radicals in the system, thereby reducing the overall molecular number. Figure 10d shows the changes in the number of different chemical species in the system, reflecting the complexity of the reaction system. The number of species fluctuates in the initial stage, followed by a gradual decline and eventual stabilization. This indicates that a certain number of intermediate species are formed in the early stages, while some are consumed or transformed as the reaction proceeds, leading to a simplified reaction network. Furthermore, the number of species under high-pressure conditions is generally higher than under low-pressure conditions, indicating more diverse reaction pathways and a greater variety of intermediate species. This observation highlights that elevated pressure not only accelerates reaction kinetics but also increases the complexity of interfacial chemical processes.

Figure 11 illustrates the temporal evolution of C–O and C–H bond populations during CMP in an H_2_O_2_ environment. Figure 11a shows a marked difference between the evolution of these bond structures in an H_2_O_2_ environment and those in the •OH environment. Under 10 MPa, the C–O bond population increases noticeably during the initial stage (0–10 ps), indicating that even under low-pressure conditions, the small amount of reactive oxygen species generated by H_2_O_2_ decomposition can still promote the initial oxidation reaction on the surface. In the subsequent equilibration stage (10–60 ps), the number of C–O bonds increases to approximately 30 and then remains relatively stable with small fluctuations, indicating that the system has entered a relatively stable reaction state, and the oxidation process is limited by the interfacial contact conditions and the availability of reactive species. Upon entering the sliding stage (60–150 ps), the number of C–O bonds exhibits pronounced fluctuations after a brief period of stability and subsequently shows an overall increasing trend, reaching a maximum of approximately 75. This phenomenon indicates that shear-induced activation enhances interfacial reactivity, allowing more newly exposed carbon atoms to participate in the oxidation reaction. The significant fluctuations further suggest that the C–O bonds undergo dynamic formation and breakage processes under the combined effects of shear stress and limited reactant supply. Under 100 MPa, the C–O bond population increases rapidly in the initial stage and then remains stable at approximately 30–40 throughout the process, with relatively minor fluctuations. This indicates that although high pressure enhances interfacial contact and accelerates initial oxidation, the oxidation reaction is difficult to further progress due to limited generation of active species from H_2_O_2_ decomposition, thereby resulting in a plateau-like characteristic in the number of C–O bonds. Figure 11b shows that the evolution trend of C–H bonds in the H_2_O_2_ system exhibits characteristics distinctly different from those in the •OH environment. Under 10 MPa, C–H bonds are nearly absent during the initial stage but gradually increase during the equilibration stage, and increase in the sliding stage (60–150 ps), showing an overall continuous growth trend. This result indicates that in relatively weak oxidation conditions, some unoxidized surface carbon atoms are more likely to combine with the hydrogen source, thereby forming C–H bonds. Furthermore, the large fluctuations indicate that C–H bonds continuously undergo formation and breakage processes under shear, reflecting their relatively low stability. In contrast, the C–H bond population under 100 MPa conditions remains at an extremely low level throughout the simulation process, increasing only slightly in the later stages. This behavior indicates that although the overall oxidation capacity of the H_2_O_2_ system is limited, the enhanced interfacial reactions under high pressure preferentially consume active sites, making them more likely to participate in oxidation rather than hydrogenation reactions, thereby inhibiting the formation of C–H bonds. Combining the evolution of C–O and C–H bonds, these results indicate that interfacial reactions in an H_2_O_2_ environment are limited by the generation rate of active species, and the bond structure evolution reflects a competitive relationship between oxidation and hydrogenation. Compared with the •OH oxidation system, the bond structure evolution in the H_2_O_2_ system is more moderate. These findings suggest that diamond surface reactions in an H_2_O_2_ environment tend toward controlled surface modification rather than drastic structural destruction.

### 3.3. Analysis of Experimental Results

ReaxFF MD simulations demonstrate that high-pressure H_2_O_2_ environments enhance chemical interactions between the polishing slurry and the diamond surface, thereby promoting surface oxidation and facilitating subsequent material removal processes. These findings highlight the critical role of pressure-enhanced interfacial reactions in promoting surface oxidation and activation during CMP. Nevertheless, it should be noted that atomic-scale simulations are inherently constrained by limited temporal and spatial scales, which limits their ability to fully capture the complexity of practical CMP processes. Consequently, the reliability and applicability of the simulated results require further validation through macroscopic experiments. Accordingly, diamond CMP experiments are conducted in this section in an H_2_O_2_ environment, aiming to elucidate the relationship between interfacial reaction dynamics and surface oxidation behavior, and to validate the mechanistic insights derived from ReaxFF simulations.

Figure 12 presents the XPS characterization results of the diamond surface before and after CMP using SiO_2_ abrasives within a high-pressure H_2_O_2_ slurry system, aiming to elucidate the evolution of the surface chemical state during polishing. As shown in Figure 12a, the pristine diamond surface exhibits a prominent C1s peak centered at 284.8 eV, indicating a typical structure of intrinsic carbon. The absence of detectable oxygen-related signals suggests a chemically stable surface with a negligible degree of oxidation, confirming that the initial diamond surface has not undergone any significant oxidation or chemical modification. In contrast, the XPS survey spectrum after CMP reveals that a pronounced O1s signal emerges at approximately 531.5 eV in addition to the dominant C1s peak, indicating the incorporation of oxygen species onto the diamond surface during polishing and implying the occurrence of interfacial oxidation reactions [77]. Although SiO_2_ abrasives can partially remove the oxide layer formed on the surface, their mechanical removal capacity is relatively limited. As a result, some oxidation products remain on the diamond surface in the form of oxygen-containing functional groups, manifesting as a detectable O1s signal in XPS spectra, as observed in Figure 12b. After CMP, changes in the C 1s spectral profile indicate modification of the near-surface carbon chemical environment. However, changes in the survey-peak intensity alone are insufficient to establish the formation of sp^2^ or amorphous carbon. Therefore, the present XPS analysis is primarily used to evaluate surface chemical-state changes and the formation of oxygen-containing functional groups. Meanwhile, the XPS survey spectrum after CMP is deconvoluted into distinct components corresponding to O–H bonds (hydroxyl groups) at approximately 530.01 eV, C=O double bonds (carbonyl groups) at 531.96 eV, and C–O single bonds at 535.18 eV, as illustrated in Figure 12c,d. These results indicate that the diamond surface undergoes a multi-step oxidation process involving adsorption, activation, and subsequent structural reconstruction, leading to the formation of diverse oxygen-containing functional groups. Specifically, the presence of hydroxyl groups indicates that the reactive intermediate species generated from H_2_O_2_ decomposition participate in the interface reaction, while the formation of carbonyl groups suggests that a more significant oxidation occurs in the localized regions. The surface roughness after polishing was measured using atomic force microscopy, and the results are shown in Figure 13. An ultra-smooth surface (Sa = 0.256 nm) was obtained within a 5 μm × 5 μm area. Roughness values of Ra = 0.239 nm and Ra = 0.407 nm were obtained in the A-A direction (parallel to the grooves) and the B-B direction (perpendicular to the grooves), respectively, demonstrating the significant polishing effect of the employed polishing fluid and pressure on the diamond surface.

## 4. Conclusions

To elucidate the interfacial oxidation behavior associated with SiO_2_-assisted chemical–mechanical polishing of single-crystal diamond, ReaxFF MD simulations were employed to investigate the evolution of surface oxidation, charge redistribution, chemical species, and bond structures under different oxidizing environments and pressure conditions. Complementary CMP experiments in an H_2_O_2_-based slurry were conducted, and XPS characterization was used to provide qualitative experimental evidence for the formation of oxygen-containing surface species predicted by the simulations. The main conclusions are summarized as follows:

(1) Oxidant chemistry and mechanical loading jointly regulate the kinetics and pathway selectivity of interfacial reactions during diamond CMP. The •OH and H_2_O_2_ systems exhibit distinct reaction characteristics because of differences in their reactive-species generation mechanisms. Increasing pressure enhances interfacial contact and promotes the activation of surface carbon sites, thereby affecting the initiation and subsequent evolution of interfacial oxidation reactions.
(2) In the •OH system, highly reactive radicals can directly interact with mechanically activated carbon sites, resulting in relatively rapid and dynamically fluctuating oxidation reactions. In contrast, oxidation in the H_2_O_2_ system is additionally governed by the stepwise decomposition of H_2_O_2_ and the generation of reactive intermediates. This decomposition-controlled process imposes an intrinsic kinetic limitation on the interfacial reaction and leads to a comparatively moderated oxidation pathway.
(3) The effect of pressure is more pronounced in the early-stage activation and reaction kinetics than in the final or instantaneous C–O bond population. Elevated pressure promotes interfacial contact, charge redistribution, reactant consumption, and initial bond activation. However, the subsequent evolution of oxygen-containing bonds is also controlled by oxidant-specific reaction kinetics, reactive-species availability, and dynamic bond formation and breakage.
(4) Interfacial oxidation preferentially occurs through interactions between reactive species and mechanically activated or localized high-energy carbon sites, accompanied by the formation and dynamic evolution of C–O, C–OH, and C–H structures. Sliding motion continuously exposes new reactive sites and enhances reactant–surface interactions, thereby coupling mechanical activation with surface chemical reactions. XPS measurements after H_2_O_2_-based CMP confirm the presence of oxygen-containing functional groups on the diamond surface, providing qualitative experimental support for the occurrence of interfacial oxidation.

## Figures and Tables

**Figure 1:**
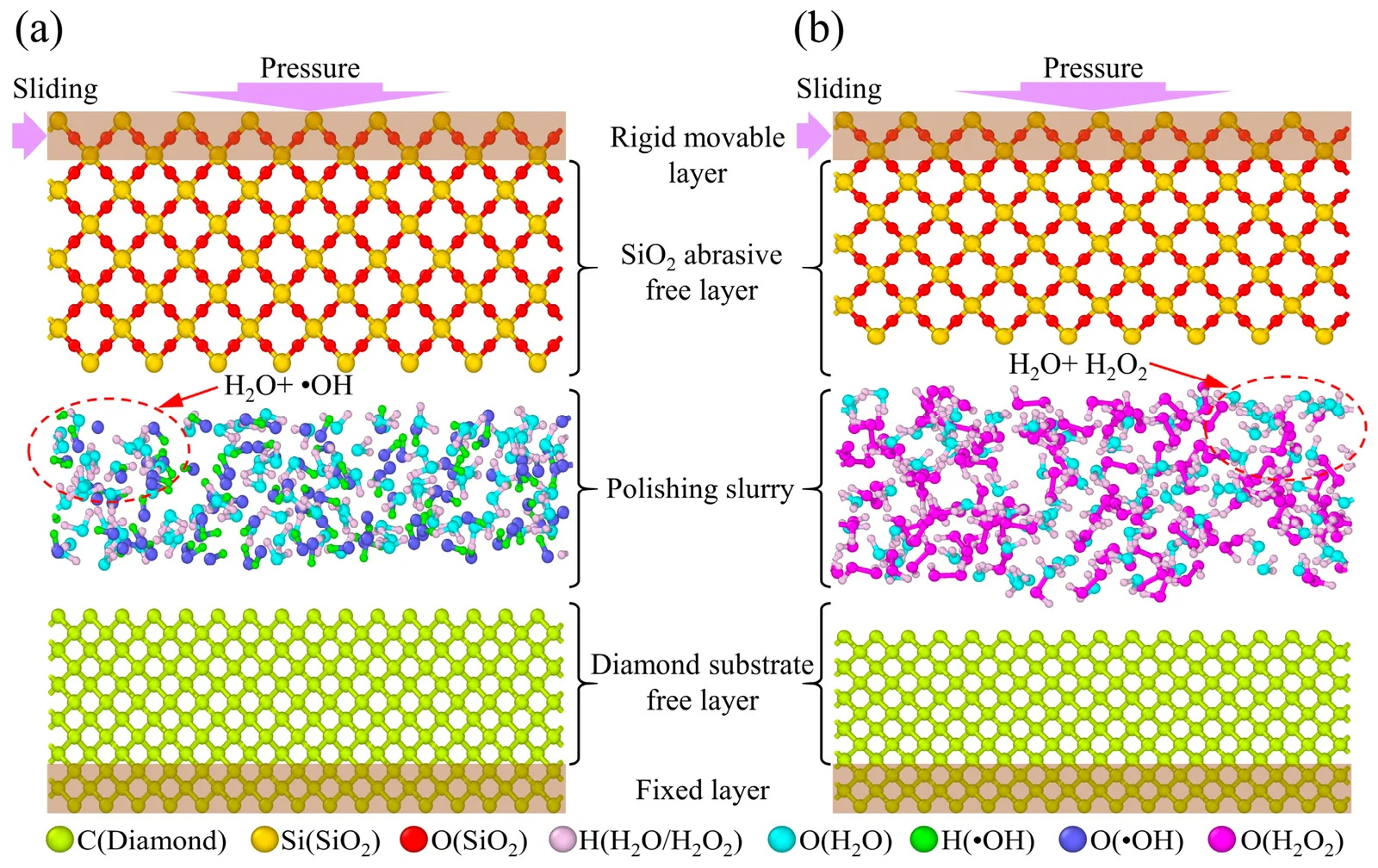
ReaxFF MD model of diamond CMP under different polishing environments. (**a**) •OH environment, (**b**) H_2_O_2_ environment.

**Figure 2:**
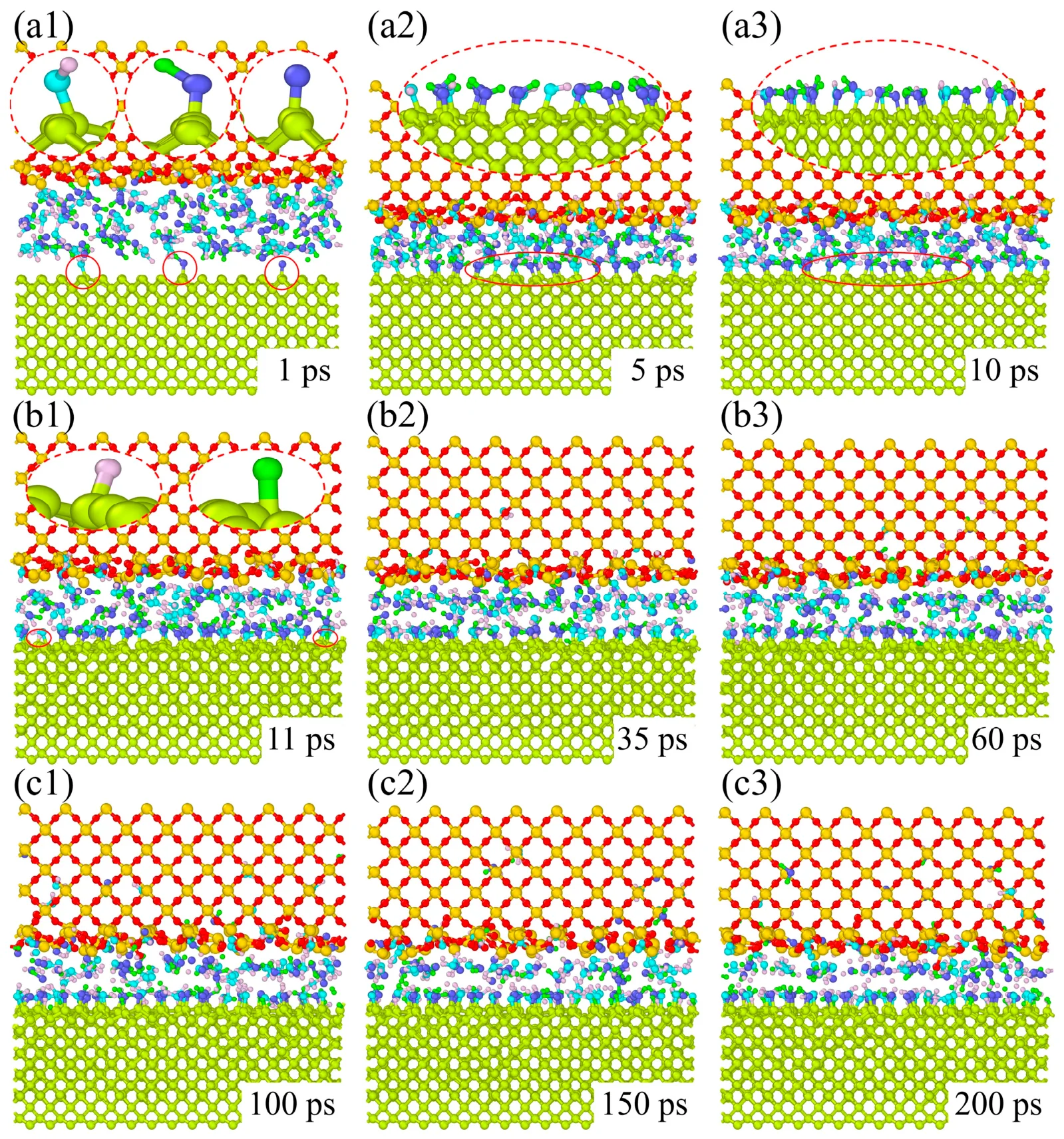
Evolution of diamond surface oxidation under 10 MPa in •OH environment. (**a1**–**a3**) Pressure stage, (**b1**–**b3**) equilibration stage, and (**c1**–**c3**) sliding stage. The red circles indicate locally enlarged views of surface carbon atoms.

**Figure 3:**
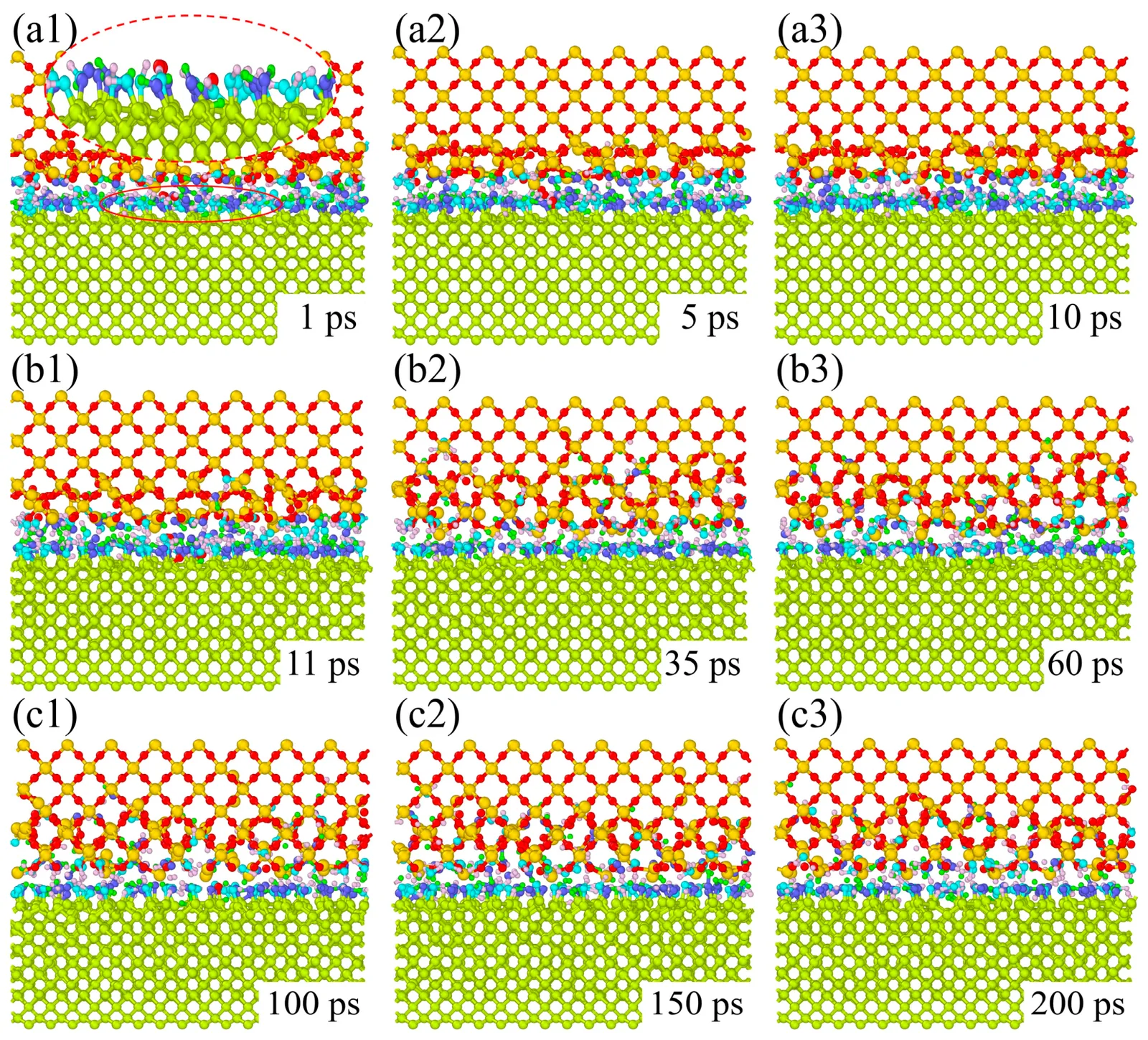
Evolution of diamond surface oxidation under 100 MPa in •OH environment. (**a1**–**a3**) Pressure stage, (**b1**–**b3**) equilibration stage, and (**c1**–**c3**) sliding stage. The red circles indicate locally enlarged views of surface carbon atoms.

**Figure 4:**
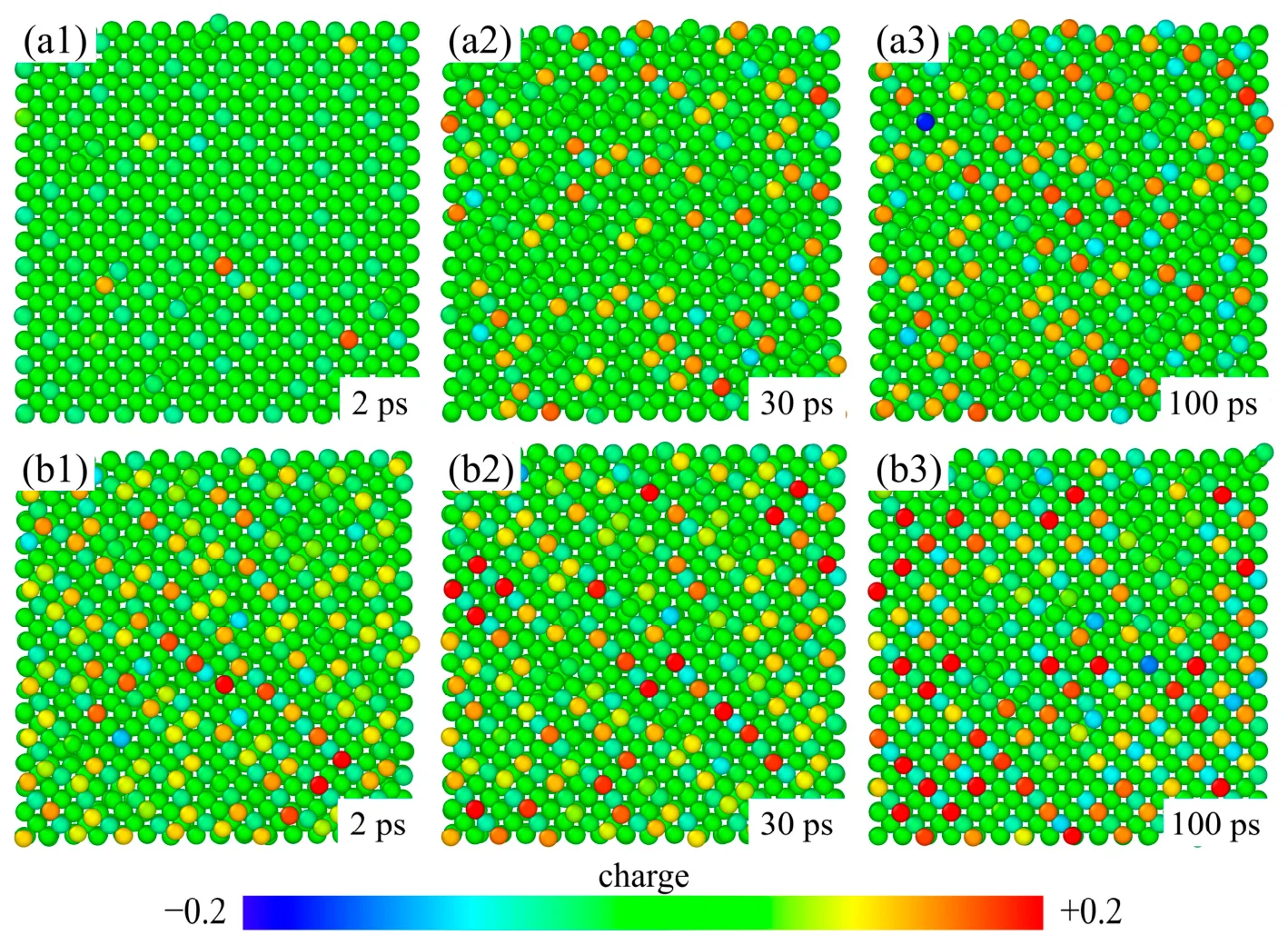
Evolution of charge distribution on diamond surfaces under varying pressure conditions in •OH environment. (**a1**–**a3**) 10 MPa, (**b1**–**b3**) 100 MPa.

**Figure 5:**
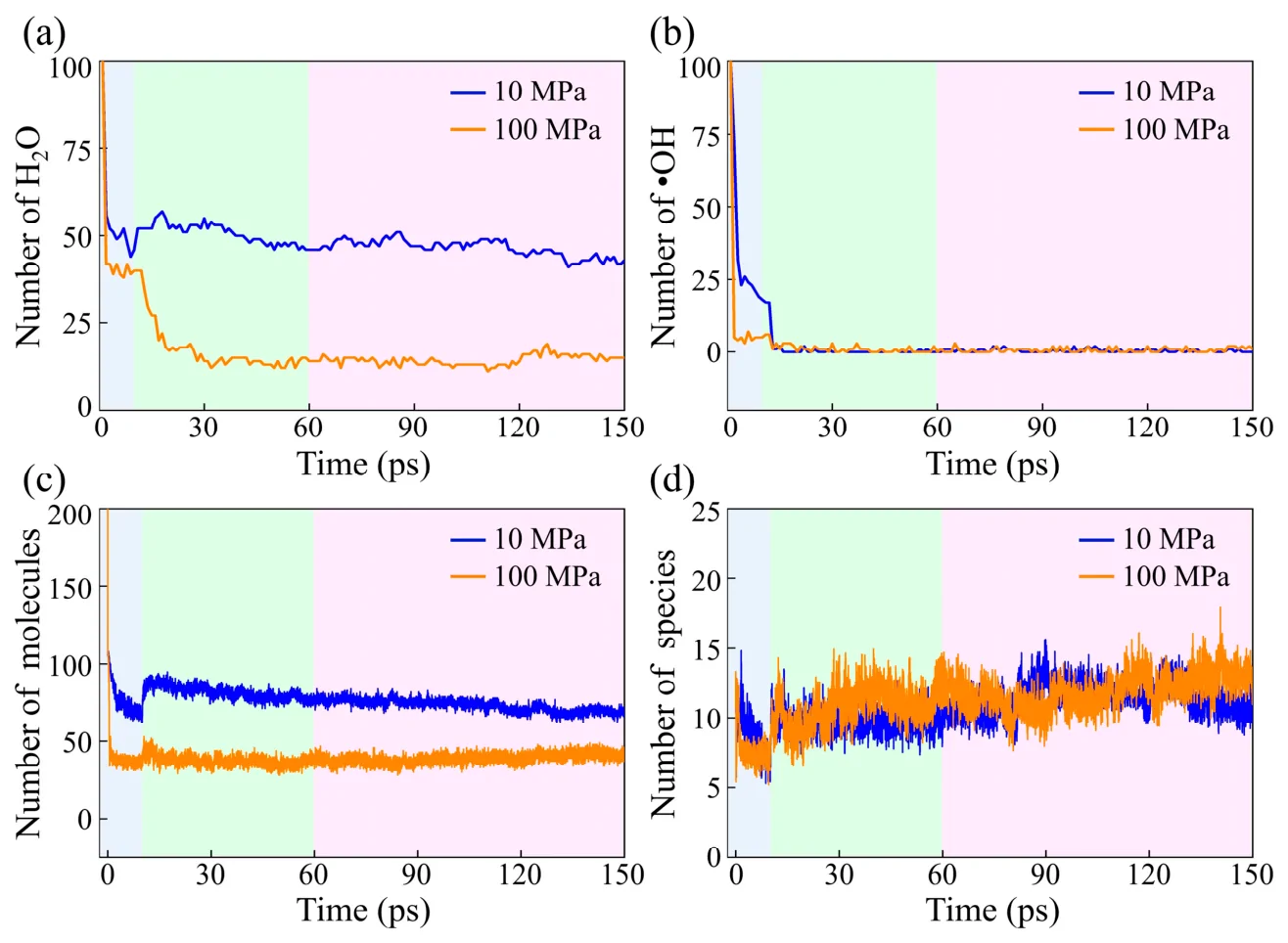
Evolution of (**a**) H_2_O, (**b**) •OH radicals, (**c**) molecules, and (**d**) species under varying pressure conditions in •OH environment. The blue, green, and pink regions represent the pressure, equilibration, and sliding stages, respectively.

**Figure 6:**
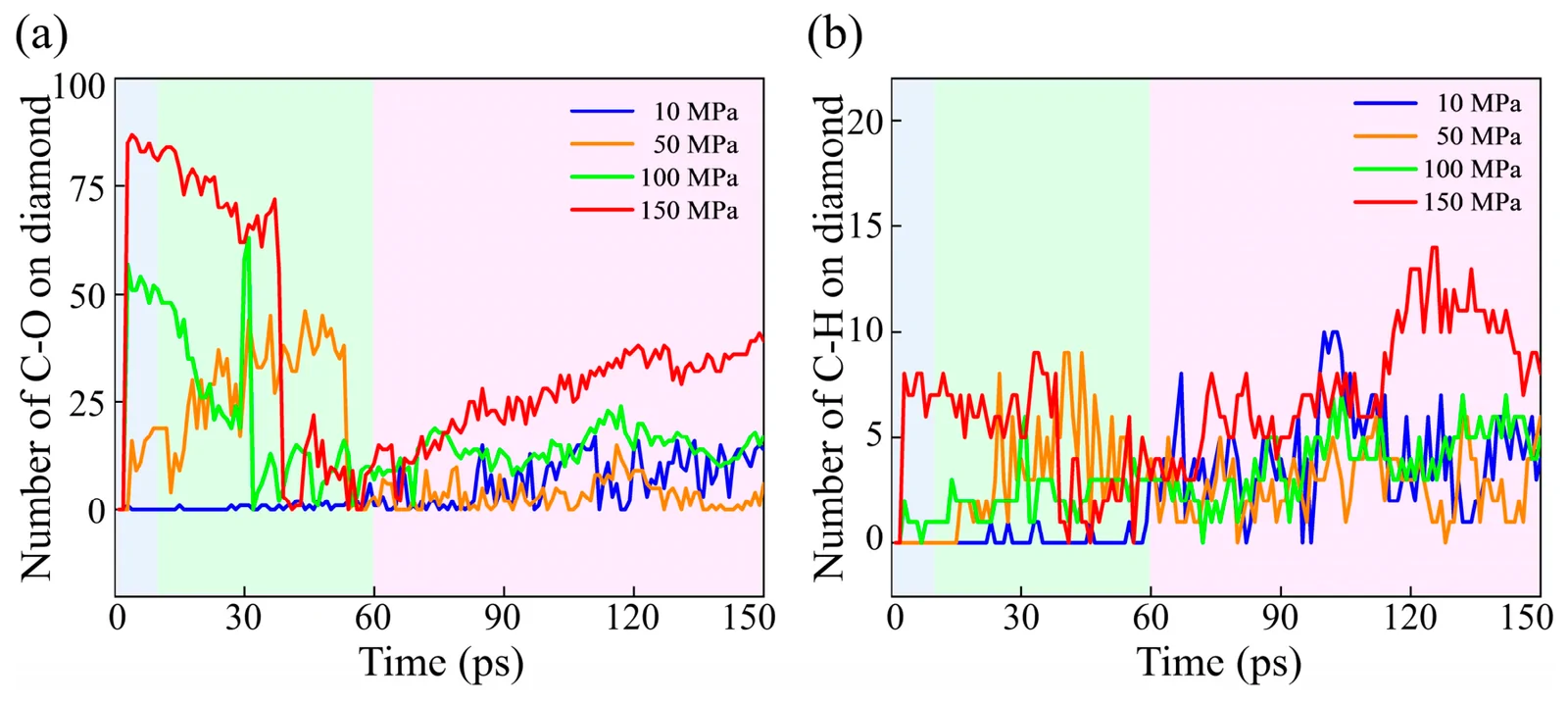
Evolution of diamond surface structure under varying pressure conditions in •OH environment. (**a**) C–O, (**b**) C–H. The blue, green, and pink regions represent the pressure, equilibration, and sliding stages, respectively.

**Figure 7:**
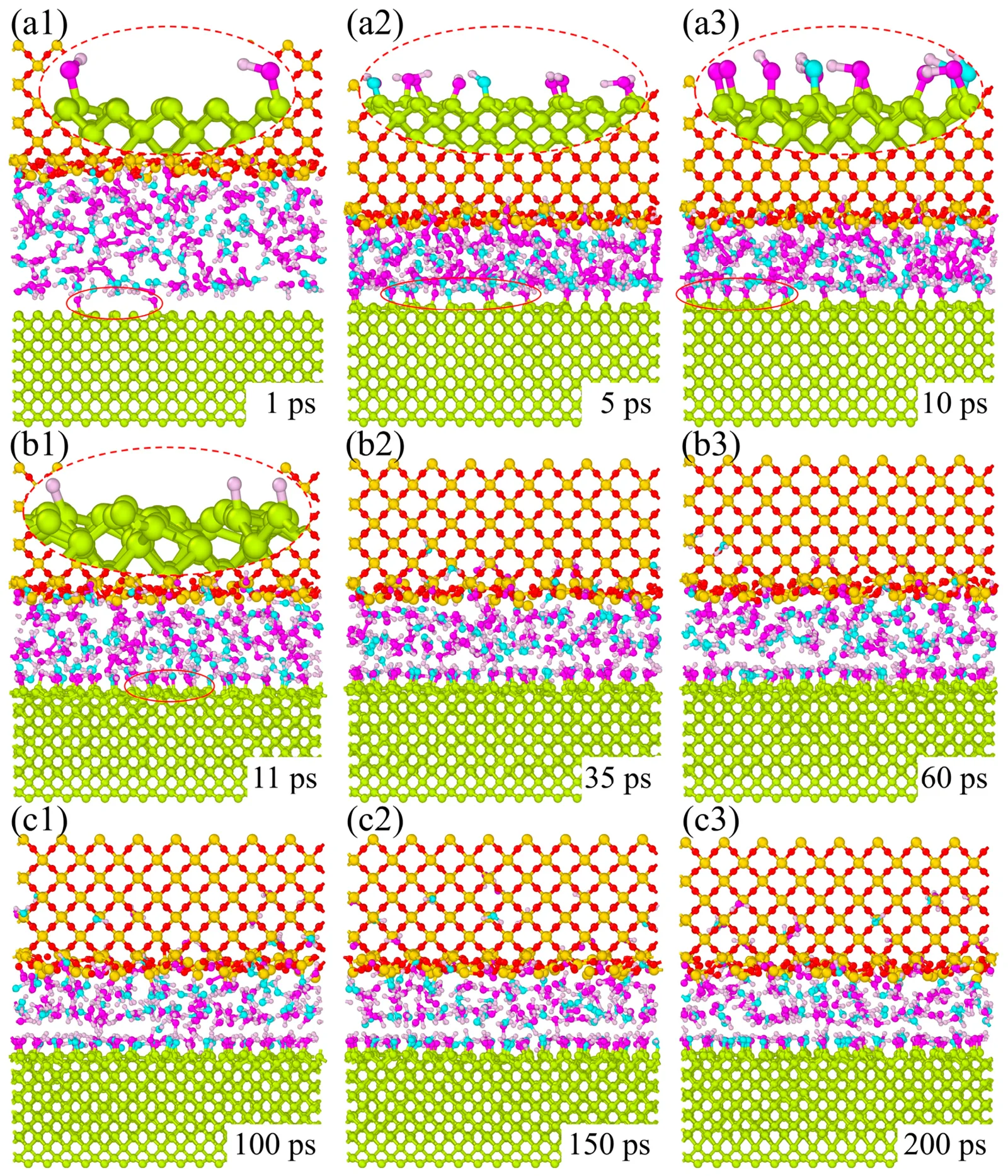
Evolution of diamond surface oxidation under 10 MPa in H_2_O_2_ environment. (**a1**–**a3**) Pressure stage, (**b1**–**b3**) equilibration stage, and (**c1**–**c3**) sliding stage. The red circles indicate locally enlarged views of surface carbon atoms.

**Figure 8:**
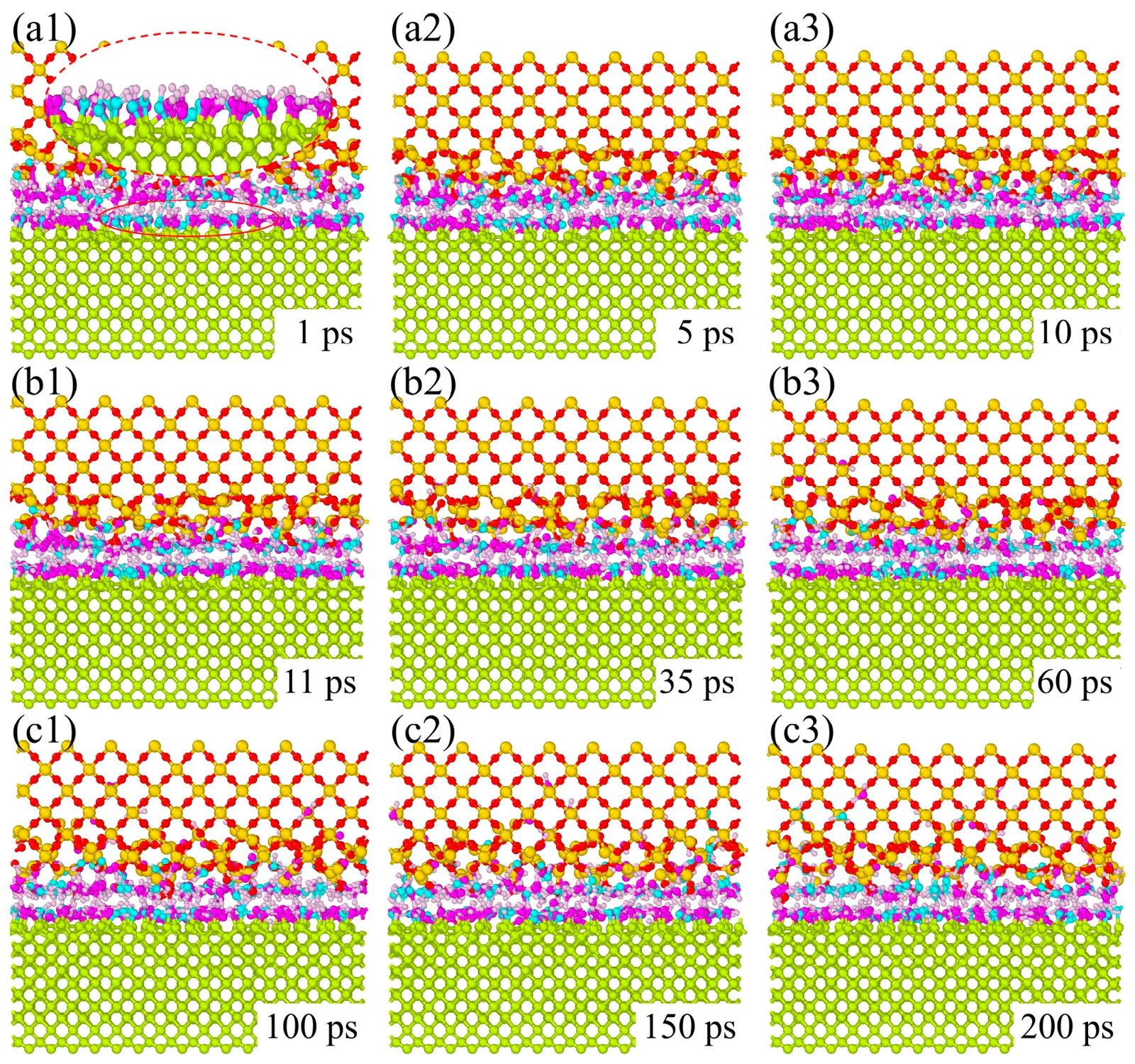
Evolution of diamond surface oxidation under 100 MPa in H_2_O_2_ environment. (**a1**–**a3**) Pressure stage, (**b1**–**b3**) equilibration stage, and (**c1**–**c3**) sliding stage. The red circles indicate locally enlarged views of surface carbon atoms.

**Figure 9:**
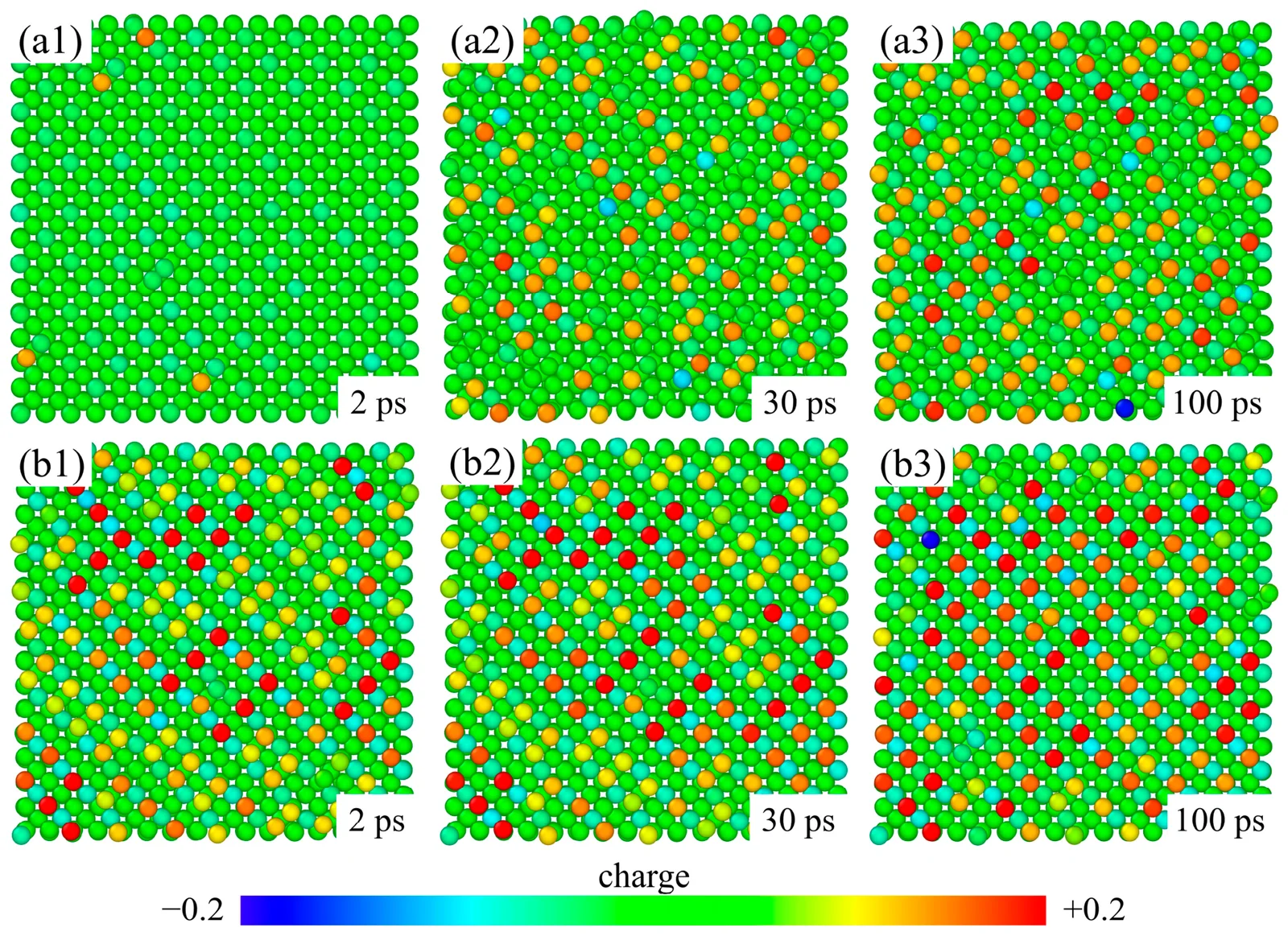
Evolution of charge distribution on diamond surfaces under varying pressure conditions in H_2_O_2_ environment. (**a1**–**a3**) 10 MPa, (**b1**–**b3**) 100 MPa.

**Figure 10:**
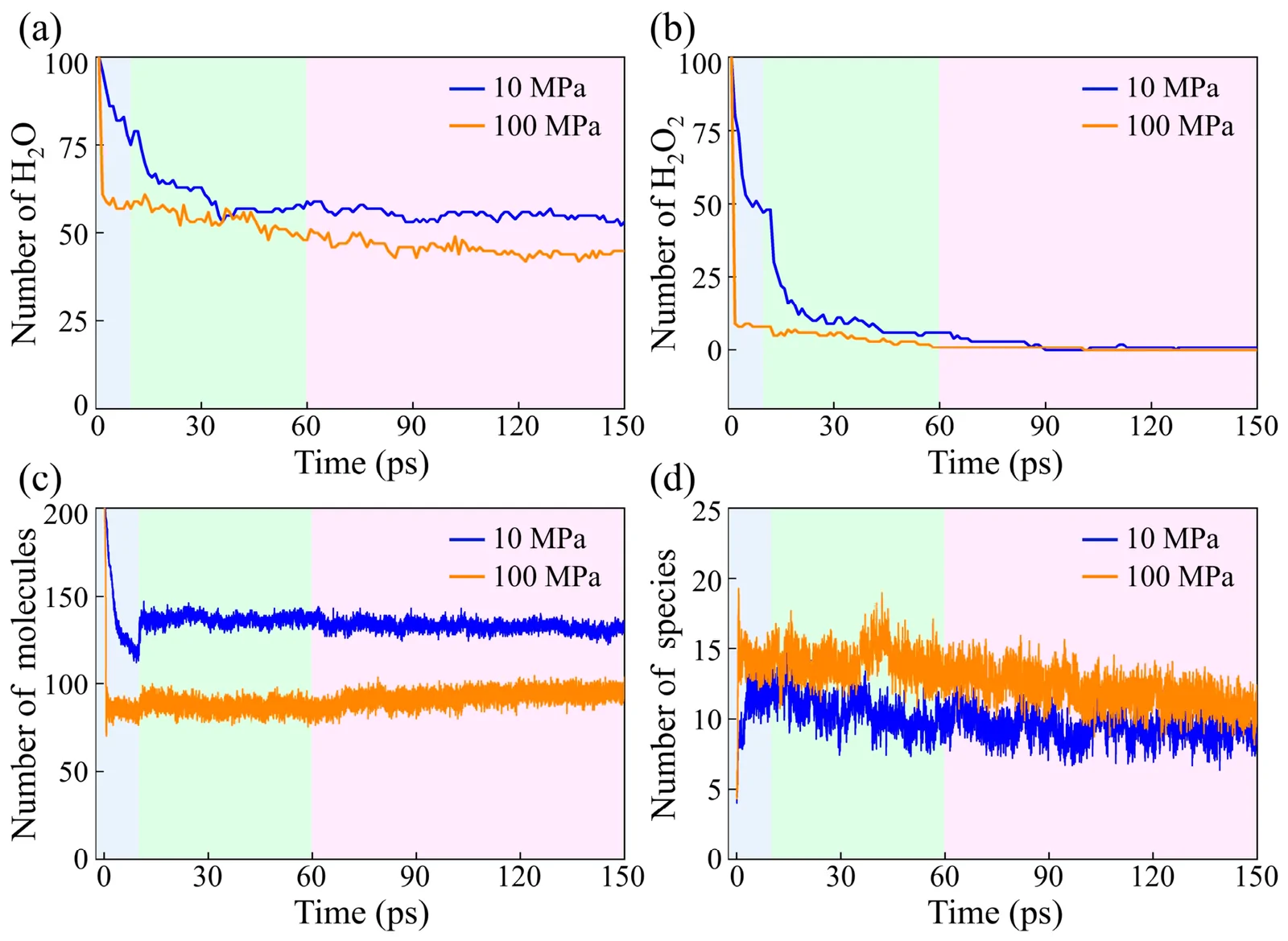
Evolution of (**a**) H_2_O, (**b**) H_2_O_2_, (**c**) molecules, and (**d**) species under varying pressure conditions in H_2_O_2_ environment. The blue, green, and pink regions represent the pressure, equilibration, and sliding stages, respectively.

**Figure 11:**
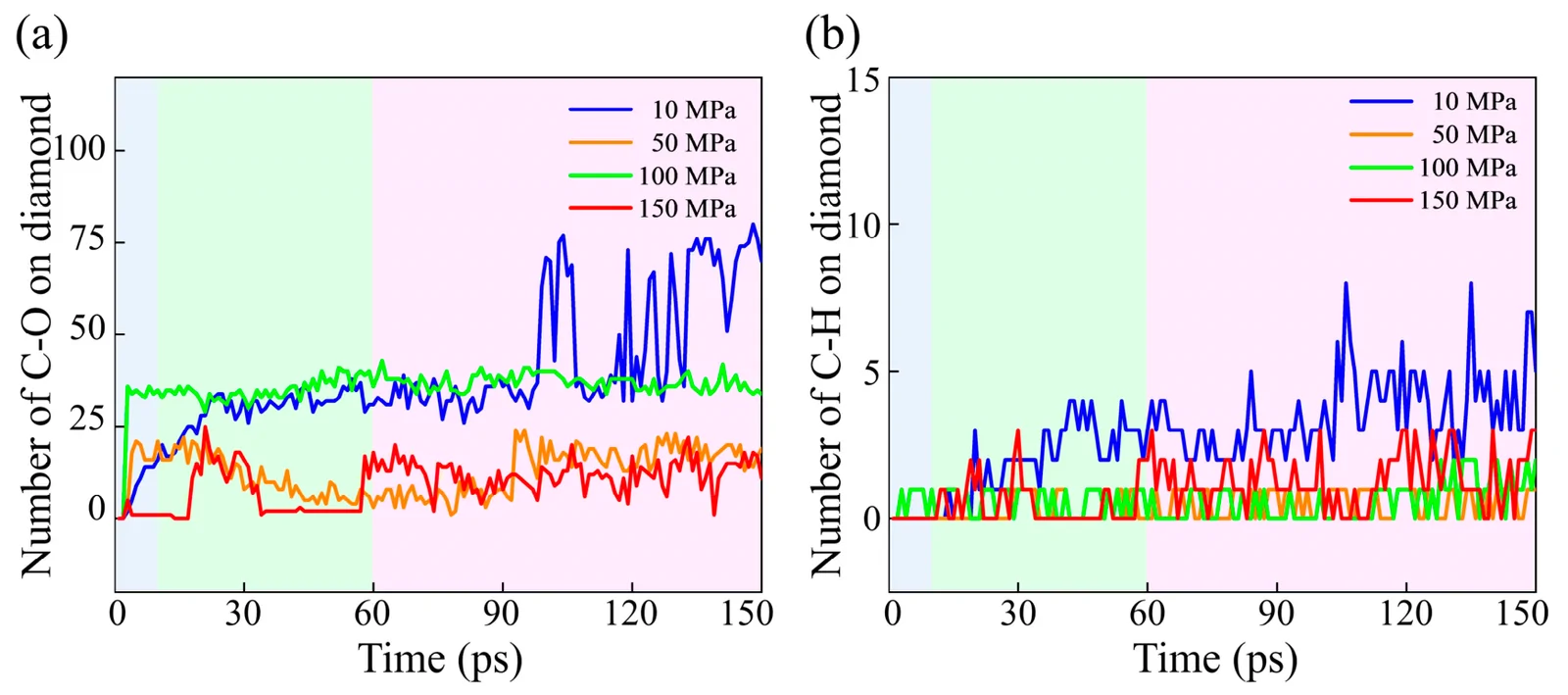
Evolution of diamond surface structure under varying pressure conditions in H_2_O_2_ environment. (**a**) C–O, (**b**) C–H. The blue, green, and pink regions represent the pressure, equilibration, and sliding stages, respectively.

**Figure 12:**
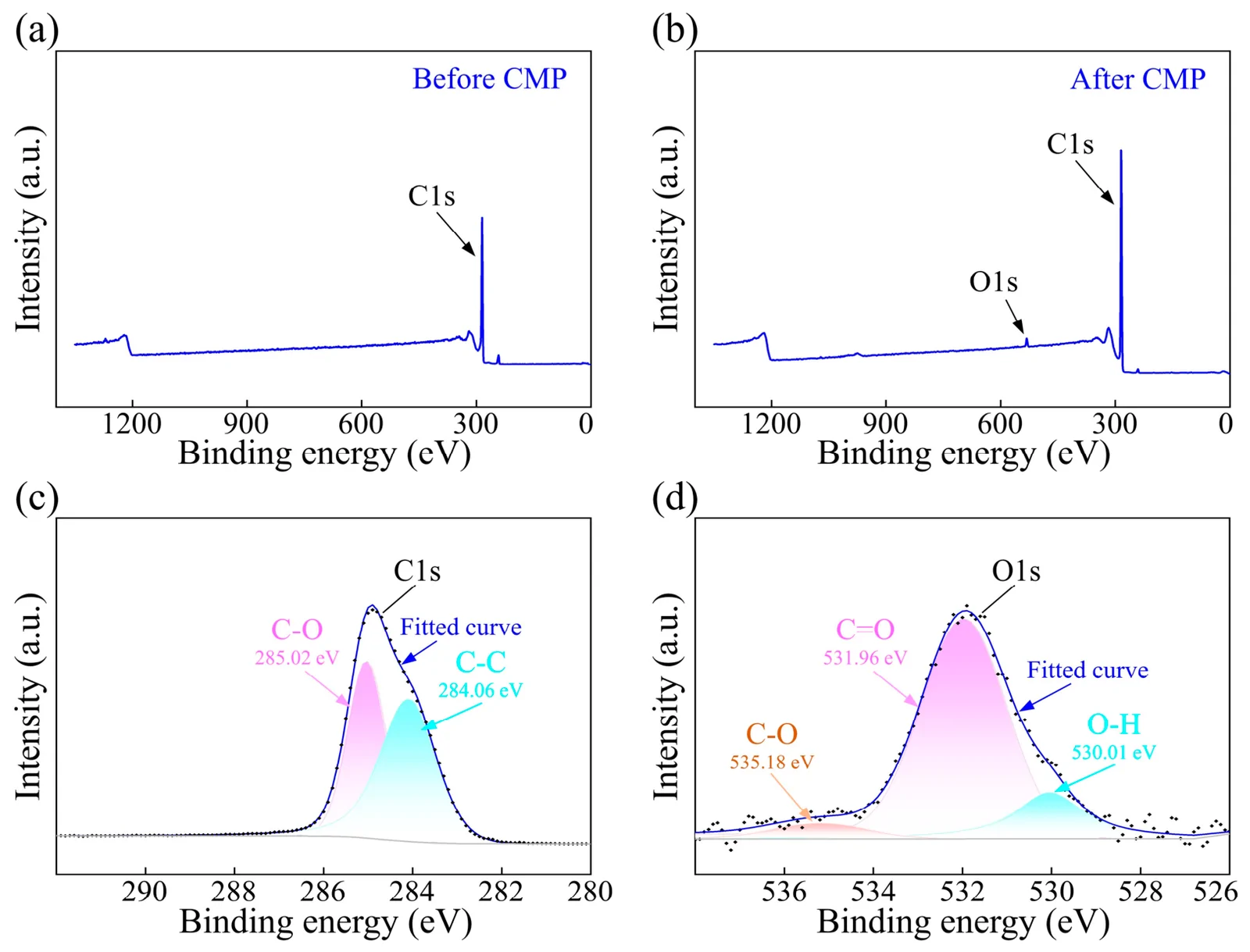
XPS survey and high-resolution spectra of the diamond surface. (**a**) Survey spectrum before CMP, (**b**) survey spectrum after CMP, (**c**) C1s spectrum after CMP for 1 h, and (**d**) O1s spectrum after CMP for 1 h. For (**c**,**d**), the blue dots represent the experimental data, while the blue solid lines denote the fitted curves.

**Figure 13:**
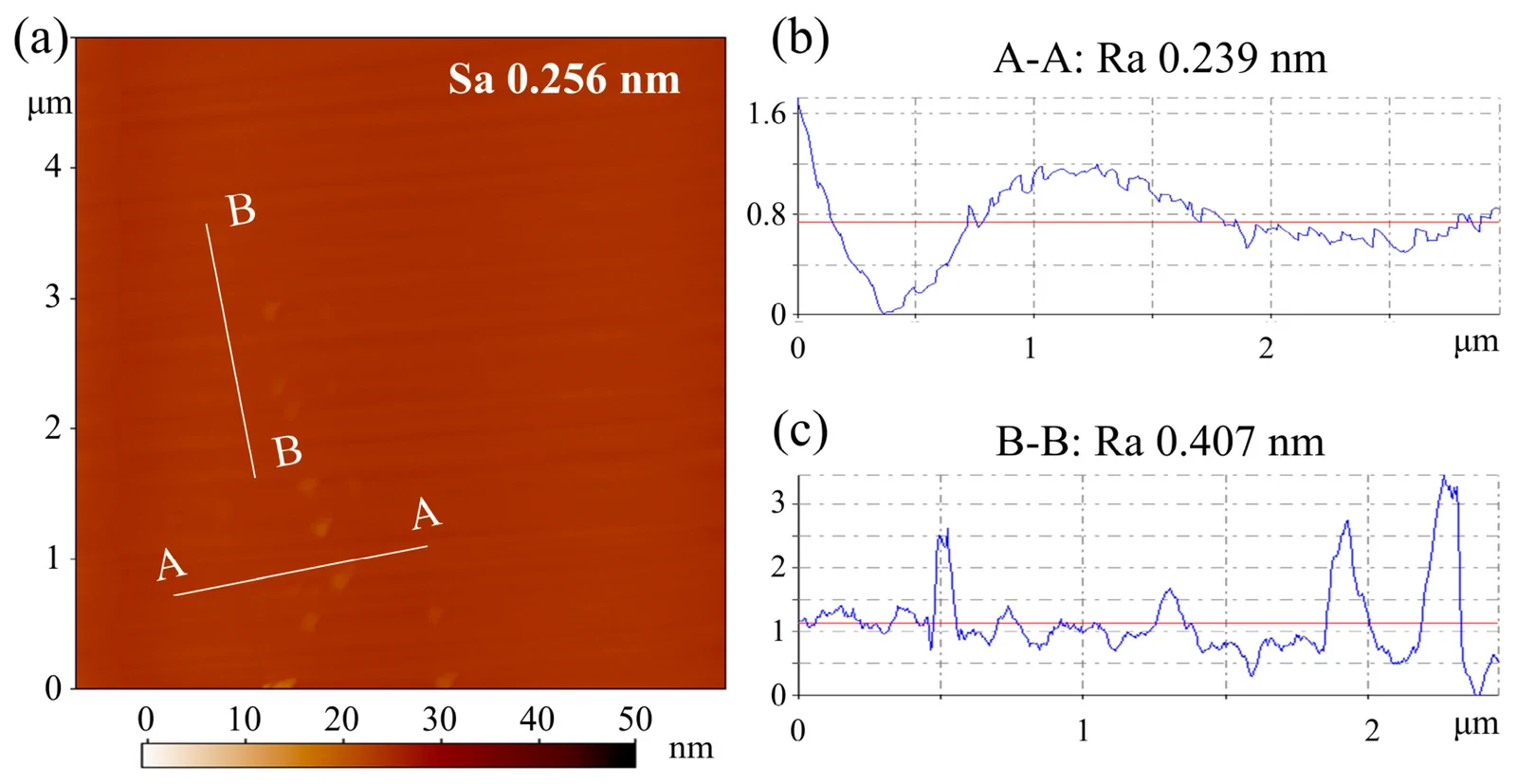
AFM measurement results after 1 h of polishing. (**a**) AFM image of the measured surface area, (**b**) surface profile along the A–A direction (parallel to the grooves), and (**c**) surface profile along the B–B direction (perpendicular to the grooves). The red horizontal line denotes the mean profile line, which serves as the reference for calculating the profile roughness.

**Table 1:** Detailed parameter settings in the ReaxFF MD simulation.

Parameters	Values
Substrate material	single-crystal diamond (100) plane
Abrasive material	SiO_2_
Directions (x × y × z)	[100]/[010]/[001]
Dimensions (Å^3^)	28.4 × 28.4 × 45.4
Atom number of diamond	1536 C atoms
Atom number of SiO_2_	256 Si atoms and 448 O atoms
Polishing slurry	H_2_O + •OH/H_2_O + H_2_O_2_
Pressure (MPa)	10, 50, 100, 150
Boundary condition	p p s
Equilibration temperature (K)	300
Potential function	ReaxFF
Ensemble	NVT/NVE
Sliding time (ps)	150
Sliding velocity (m/s)	10
Time step (fs)	0.1

**Table 2:** Polishing parameter settings.

Parameters	Values
Sample material	CVD single-crystal diamond
Sample size (mm)	10 × 10 × 1
Crystal plane	(100) plane
Temperature (°C)	25
Load (N)	30
Rotation speed of the holder (r/min)	60
Rotation speed of the polishing disk (r/min)	80
Abrasive type	SiO_2_ particles
Abrasive diameter (nm)	50
Slurry flow rate (mL/min)	15
Frequency of reciprocating motion(/min)	3
Time (h)	1

## Data Availability

The original contributions presented in this study are included in the article. Further inquiries can be directed to the corresponding author.
